# Comprehensive Assessment of Anaplastic Lymphoma Kinase in Localized and Metastatic Prostate Cancer Reveals Targetable Alterations

**DOI:** 10.1158/2767-9764.CRC-21-0156

**Published:** 2022-05-02

**Authors:** Radhika A. Patel, Ilsa Coleman, Martine P. Roudier, Eric Q. Konnick, Brian Hanratty, Ruth Dumpit, Jared M. Lucas, Lisa S. Ang, Jin-Yih Low, Maria S. Tretiakova, Gavin Ha, John K. Lee, Lawrence D. True, Angelo M. De Marzo, Peter S. Nelson, Colm Morrissey, Colin C. Pritchard, Michael C. Haffner

**Affiliations:** 1 Division of Human Biology, Fred Hutchinson Cancer Research Center, Seattle, Washington.; 2 Department of Urology, University of Washington, Seattle, Washington.; 3 Department of Laboratory Medicine and Pathology, University of Washington, Seattle, Washington.; 4 The Brotman Baty Institute for Precision Medicine, Seattle, Washington.; 5 Division of Clinical Research, Fred Hutchinson Cancer Research Center, Seattle, Washington.; 6 Sidney Kimmel Comprehensive Cancer Center, Johns Hopkins University School of Medicine, Baltimore, Maryland.; 7 Department of Pathology, Johns Hopkins University School of Medicine, Baltimore, Maryland.

## Abstract

Anaplastic lymphoma kinase (ALK) is a tyrosine kinase with genomic and expression changes in many solid tumors. ALK inhibition is the first-line therapy for lung cancers with *ALK* alterations, and an effective therapy in other tumor types, but has not been well-studied in prostate cancer. Here, we aim to delineate the role of ALK genomic and expression changes in primary and metastatic prostate cancer. We determined ALK expression by IHC and RNA sequencing, and genomic alterations by NGS. We assessed functional consequences of ALK overexpression and pharmacologic ALK inhibition by cell proliferation and cell viability assays. Among 372 primary prostate cancer cases, we identified one case with uniformly high ALK protein expression. Genomic analysis revealed a novel *SLC45A3-ALK* fusion which promoted oncogenesis in *in vitro* assays. We observed ALK protein expression in 5 of 52 (9%) of metastatic prostate cancer cases, of which 4 of 5 had neuroendocrine features. ALK-expressing neuroendocrine prostate cancer had a distinct transcriptional program, and earlier disease progression. An ALK-expressing neuroendocrine prostate cancer model was sensitive to pharmacologic ALK inhibition. In summary, we found that ALK overexpression is rare in primary prostate cancer, but more frequent in metastatic prostate cancers with neuroendocrine differentiation. Furthermore, *ALK* fusions similar to lung cancer are an occasional driver in prostate cancer. Our data suggest that ALK-directed therapies could be an option in selected patients with advanced prostate cancer.

**Significance:**

Anaplastic lymphoma kinase (ALK) is a validated drug target in cancer. Here we delineate the spectrum of ALK alterations in prostate cancer. We show that ALK overexpression is present in advanced prostate cancers, in particular in cases with features of neuroendocrine carcinoma. Furthermore, ALK expression is associated with responses to pharmacologic ALK inhibition. Our study demonstrates that ALK-directed therapies should be considered in selected prostate cancer cases.

## Introduction

Anaplastic lymphoma kinase (ALK) is a receptor tyrosine kinase that was originally identified as a rearranged gene in anaplastic large cell lymphoma (1). Subsequently, recurrent constitutive activation of ALK through gene rearrangements or gain-of-function mutations has been documented in numerous cancer types including non–small cell lung cancer, inflammatory myofibroblastic tumor, anaplastic large cell lymphoma, diffuse large B-cell lymphoma, neuroblastoma, breast, colorectal, and renal cell carcinomas (2–5). ALK hyperactivity is a strong oncogenic driver and ALK inhibition in tumors with *ALK* gain-of-function alterations has been shown to result in profound therapeutic responses (6–9). To exploit this therapeutic vulnerability, a number of highly potent small-molecule ALK inhibitors were developed and approved as first-line therapies for *ALK*-rearranged lung cancers (10).

Given the proven efficacy of ALK inhibitors in non–small cell lung cancers, their clinical utility has been tested across a broad spectrum of tumor types. Collectively, the experience from these studies suggests that ALK represents a clinically actionable target in selected patients with ALK alterations irrespective of tumor type and cell lineage (4, 9, 11, 12).

Despite major advances in the treatment for prostate cancer, most patients with advanced metastatic prostate cancer develop resistance to current treatment modalities, which include androgen deprivation therapies and taxane-based chemotherapies. It is increasingly recognized that about 10% to 20% of advanced treatment-refractory prostate cancer exhibit histologic and molecular characteristics that are divergent from conventional prostatic adenocarcinoma (13). These features include changes in tumor cell morphology, loss of markers expressed in prostate epithelial cells, and gain of neuronal and neuroendocrine expression programs. Such neuroendocrine prostate cancers (NEPC) show a highly aggressive clinical course and there are limited therapeutic options for treating this variant of advanced prostate cancer (13, 14, 15). Therefore, there is a heightened interest to define relevant therapeutic targets and to develop novel treatments for NEPC.

A recent case report demonstrated a clinical response to the second-generation ALK inhibitor alectinib in a patient with *de novo* NEPC harboring an *ALK* p.F1174C activating mutation, suggesting that targeting ALK could be relevant in selected patients with prostate cancer (16). However, little is known about ALK alterations in a broader spectrum of prostate cancers.

Here we comprehensively investigate the expression of ALK in localized and advanced metastatic prostate cancer. Although ALK expression was uncommon in primary prostate cancers, we identified one case with high-level ALK protein expression due to a novel structural rearrangement involving *SLC45A3* and *ALK*. In metastatic prostate cancer, we observed that ALK expression is a relatively common feature of NEPC. ALK overexpression was associated with a distinct transcriptional profile and adverse clinical outcomes in patients with advanced prostate cancer. Furthermore, ALK inhibition resulted in profound growth suppression of an ALK-positive NEPC model. Collectively, our data suggest that targeting ALK could be considered in a selected subset of patients with advanced prostate cancers.

## Materials and Methods

### Cell Lines

Human cell lines LNCaP, 22Rv1, H2228, SH-SY5Y, NCI-H660, DU-145, and PC3 were obtained from the ATCC and were grown in the respective recommended media. All cell lines were obtained after 2015. MSKCC EF1 cells (which were originally derived from the organoid line MSKCC-CaP4; ref. 17) were a gift from Dr. John K. Lee (Fred Hutchinson Cancer Research Center, Seattle, WA) and were maintained in RPMI medium supplemented with 10% FBS, 100 U/mL penicillin and 100 μg/mL streptomycin, and 4 mmol/L GlutaMAX (Thermo Fisher). HTERT immortalized PrEC cells were a gift from John T. Isaacs (Johns Hopkins University, Baltimore, MD) and were grown in keratinocyte serum‐free media (Thermo Fisher) supplemented with insulin, EGF, and bovine pituitary extract (Thermo Fisher) as described previously (18). All cell lines were maintained at 37°C with 5% CO_2_. Short tandem repeat genotyping was used to authenticate the lines and cells were confirmed to be *Mycoplasma* free using the MycoAlert Detection Kit (Lonza, LT07–418). Cells were cultured no longer than 10 passages after thawing and before experimental use.

### Patient Samples

Primary prostate cancer samples used in this study comprised two cohorts. The first was a cohort of 341 radical prostatectomy samples from the Johns Hopkins School of Medicine (Baltimore, MD) which was described previously (19). The second cohort comprised 31 radical prostatectomy samples from the University of Washington (Seattle, WA; Supplementary Table S1). Metastatic cancer samples were collected as part of the Prostate Cancer Donor Program at the University of Washington and tissue microarrays (TMA) containing 52 patients’ tissue specimens from different metastatic sites (median number of sites per patient 5, range 1–18) were constructed as described previously (Supplementary Table S2; ref. 20).

### *In Silico* Expression Analysis

Differential expression analyses of publicly available RNA sequencing (RNA-seq) data realigned to the hg38 human genome using STAR v2.7.3a were carried out in R using limma v3.40.6 with the default settings for the voom, lmFit, eBayes, and topTable functions (13, 21). Gene expression results were ranked by their limma t-statistics and used to conduct Gene Set Enrichment Analysis (GSEA; ref. 22) to determine patterns of pathway activity utilizing the curated pathways from within the MSigDBv7.2. Androgen receptor (AR) and neuroendocrine signature (NE) scores were calculated using GSVA v1.32.0 using log_2_ fragments per kilobase of transcript per million mapped reads (FPKM) val- ues as input (23). Boxplots of *ALK* gene expression were created with ggplot2 v3.2.1 and statistical comparisons between groups were assessed by Wilcoxon test with Benjamini–Hochberg multiple testing correction using the ggpubr v0.2.3 stat_compare_means function. Fusion detection was carried out using STAR-fusion v1.9.1 using default parameters (24).

### Targeted Sequencing

Targeted next-generation genomic sequencing was carried out using the UW-OncoPlex version 6 assay as described previously (25). In brief, DNA extracted from formalin-fixed, paraffin-embedded tissue was subjected to SureSelect XT capture for target enrichment and sequenced on an Illumina NextSeq 500 instrument with paired-end 101-bp reads. This validated target capture clinical-grade sequencing assay covers a total of 1.9 MB of DNA encompassing 340 genes, including all exons of *ALK* and has been validated for fusion detection, including for fusions in which only one partner is captured (25, 26). Specifically, the assay uses three separate structural variant callers, including GRIDSS, BreakDancer, and Pindel. Raw data from fusions were also manually reviewed by a panel of expert molecular pathologists. (C.C. Pritchard and E.Q. Konnick).

### IHC Staining

For IHC staining, slides were deparaffinized and steamed for either 30 minutes in 10 mmol/L sodium citrate (pH  =  6, Vector Labs) or for 45 minutes in Target Retrieval Solution (Dako). Primary antibodies and dilutions used were as follows: ALK (clone D5F3, Cell Signaling Technology, 3633T, 1:100), AR (Cell Signaling Technology, 5153T, 1:100), synaptophysin (Thermo Fisher, RM9111S, 1:80), and NKX3.1 (Thermo Fisher, 5082788, 1:50). Immunocomplexes were detected using the UltraVision Quanto Detection System with DAB as the chromogen (Thermo Fisher, TL060QHD). Tissue sections were counterstained with hematoxylin and slides were digitized on a Ventana DP 200 Slide Scanner (Roche). Immunoreactivity was scored in a blinded manner by two pathologists (M. Roudier and M.C. Haffner) whereby the optical density level (“0” for no brown color, “1” for faint and fine brown chromogen deposition, and “2” for prominent chromogen deposition) was multiplied by the percentage of cells at each staining level, resulting in a total score range of 0 to 200. The final score for each sample was the average of two duplicated tissue cores (20).

### ALK Cloning and Lentivirus Production

Lentiviral full-length ALK and the corresponding control pHAGE vectors were purchased from Addgene (Addgene IDs: 116712 and 118692). The coding sequence representative of exons 16 to 29 of *ALK* (ALK16–29) was amplified by PCR using the full-length vector as a template and was cloned into the pHAGE vector after *Xho*I digestion using the HiFi DNA Assembly Kit (New England Biolabs) following manufacturer's instructions. For lentiviral packaging, 293T cells were transfected with either pHAGE or pHAGE-ALK16–29 vectors along with packaging plasmids using calcium phosphate. Lentiviral particles were concentrated by ultracentrifugation and cells were transduced with an MOI of 2.

### Proliferation Assay

hTERT-PrEC cells were stably transduced with the pHAGE (empty vector control) and pHAGE-ALK16–29. After selection in puromycin, cells were seeded at a density of 7,000 cells/well in 96-well plates. DMSO or crizotinib (Selleckchem) was added 24 hours postseeding in triplicate wells. Cell proliferation was monitored using a Cytation5 live cell imaging instrument (BioTek). Images were acquired every 12 hours and image analysis was performed using the Gen5 software (BioTek).

### Immunoblots

Cell lysates were prepared in 1× RIPA buffer (Sigma) supplemented with phosphatase and protease inhibitors (Roche) and subsequently separated by SDS-PAGE. Proteins were transferred onto nitrocellulose membranes and probed with the following antibodies at the indicated dilutions at 4°C for 16 hours: ALK (Cell Signaling Technology, 3633T) 1:2,000, phospho-Akt (Cell Signaling Technology 4060T) 1:2,000, pan-Akt (Cell Signaling Technology 4691T) 1:1,000, and β-actin (Cell Signaling Technology 4970S) 1:1,000. Immunocomplexes were detected using horseradish peroxidase–conjugated anti-mouse or anti-rabbit secondary antibody and visualized using a ChemiDoc Imaging System (Bio-Rad).

### *In Vitro* Drug Treatments

For cell viability studies, cells were seeded at 20,000 cells/well in 96-well plates. After a 24-hour recovery, cells were treated with serial dilutions of either crizotinib (Selleckchem), ceritinib (Selleckchem), or solvent (DMSO) in triplicates. Cells were exposed to two doses of ALK inhibitors. The first dose was added 24 hours after seeding and the second dose was added 72 hours after the first dose. Viability was determined using CellTiter-Blue (Promega G8080) 48 hours following the second dose.

### Statistical Analyses

Statistical analyses for *in vitro* data were performed using GraphPad Prism 7 with the tests indicated in the figure legends. For single comparisons, statistical analyses were performed using a two-sided Student *t* test. Best-fit curves were generated with linear regression modeling. *P* < 0.05 was considered to indicate a statistically significant difference. Outcome analyses were performed using the survival package version 3.2–11 in R version 4.0.2. Groups were constructed based on log_2_ FPKM of *ALK* expression as determined by RNA-seq analysis. Survival differences were calculated using the survdiff method in survival (27).

### Data Availability Statement

Transcriptomic data used in this study are publicly available in Gene Expression Omnibus (GEO) at GSE158593 and GSE126078. All other data generated in this study are available upon request from the corresponding author.

## Results

### ALK Alterations are Rare in Localized Prostate Cancer

**FIGURE 1:**
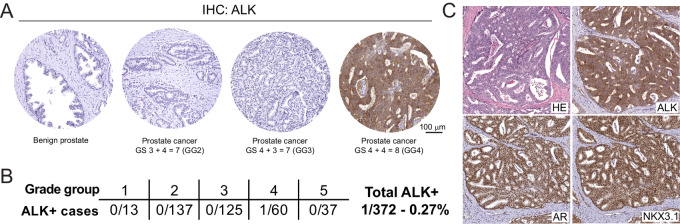
ALK protein expression in primary localized prostate cancers. **A,** ALK expression was assessed by IHC using a validated antibody (clone D5F3) in two primary prostate cancer cohorts from the Johns Hopkins Hospital (*n* = 341) and the University of Washington (*n* = 31). **B,** Out of a total of 372 cases, only one tumor was positive for ALK. **C,** Micrographs of the ALK-positive case showed cribriform morphology and expression of AR and NKX3.1 confirm the prostatic origin of this tumor.

To comprehensively assess the expression of ALK in a representative cohort of primary prostate cancers, we performed IHC staining for ALK on TMAs of radical prostatectomy specimens from two academic centers (The Johns Hopkins School of Medicine and University of Washington) using a clinical grade and extensively validated ALK antibody (clone D5F3; ref. 28; Supplementary Fig. S1A). Out of a total of 372 cases, we identified one tumor with robust ALK staining (Fig. 1A and B). The ALK-positive tumor showed a Gleason score of 4+4 = 8 with extensive large cribriform morphology (Fig. 1C). Tumor cells exhibited a uniform strong cytoplasmic immunoreactivity for ALK, expressed AR and NKX3.1 (Fig. 1C; Supplementary Fig. S1B) but were negative for the neuroendocrine marker synaptophysin (Supplementary Fig. S1B). This staining pattern confirmed the prostatic lineage origin of this lesion and the absence of neuroendocrine differentiation.

### NGS Reveals an Oncogenic *ALK* Rearrangement

**FIGURE 2:**
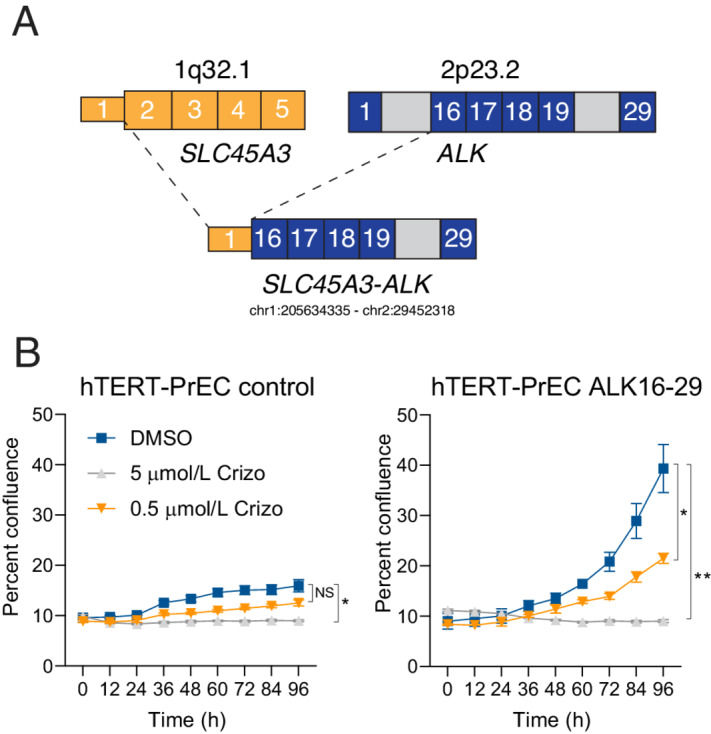
NGS of the ALK-expressing tumor reveals a novel oncogenic *SLC45A3*-*ALK* rearrangement. **A,** Schematic showing the fusion transcript involving noncoding exon 1 of *SLC45A3* (break point hg19 chr1:205634335) and exon 16 of *ALK* (break point hg19 chr2:29452318). **B,** Overexpression of the fusion transcript comprising ALK exons 16–29 (ALK16–29) in hTERT immortalized benign prostate epithelial cells (hTERT-PrEC) resulted in significantly increased cell proliferation which was reduced by treatment with the ALK inhibitor crizotinib (crizo).

To understand the underlying mechanism responsible for ALK overexpression in this case, we performed targeted sequencing using the UW-OncoPlex clinical sequencing platform (Supplementary Fig. S2). This revealed a rearrangement involving *ALK* and the androgen-regulated and prostate-specific gene *SLC45A3* (Fig. 2A; Supplementary Fig. S3) which resulted in a fusion of the 5′ untranslated region of *SLC45A3* to intron 15 of *ALK*. The predicted coding fusion transcript encompassed exons 16–29 of *ALK*, which includes its C-terminal kinase domain (Fig. 2A). *SCL45A3* is an androgen-regulated gene that, like *TMPRSS2*, is seen as a recurrent fusion partner with *ERG* in prostate cancer (29). It is important to note that in previously published cohorts, *ALK* gene alterations in localized prostate cancers appeared extremely rare and encompassed only nonpathogenic single-nucleotide variants or low-level copy-number gains (refs. 30, 31; Supplementary Fig. S4).

To understand the functional consequences of this *ALK* rearrangement, we generated lentiviral expression constructs encoding for *ALK* exons 16–29 (ALK16–29) or empty vector controls and transduced hTERT immortalized benign prostate epithelial cells (hTERT-PrEC, Fig. 2B; ref. 18). ALK16–29 overexpression resulted in profoundly increased cell proliferation compared with controls (Fig. 2B). Notably, this effect was significantly decreased in the presence of the ALK inhibitor crizotinib (Fig. 2B). Collectively, these observations suggest that the novel *SLC45A3-ALK* rearrangement that we identified likely represents a targetable oncogenic driver.

### ALK Alterations in Advanced Metastatic Prostate Cancer

**FIGURE 3:**
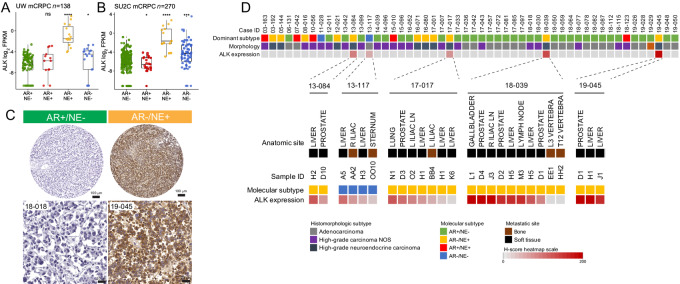
ALK is overexpressed on the mRNA and protein levels in advanced metastatic prostate cancers with neuroendocrine differentiation. *In silico* analyses of publicly available datasets from advanced castration resistant prostate cancer samples from the University of Washington (UW) rapid autopsy cohort (*n* = 138 metastases from 72 patients; **A**) and the Stand-Up To Cancer (SU2C) international dream team (*n* = 270; **B**) reveal increased ALK mRNA expression in advanced prostate cancer enriched for tumors characterized by neuroendocrine (NE+) signature expression and absence of AR signature expression (AR−; ref. 13). **C,** Representative micrographs of FFPE metastatic prostate cancer samples from the UW rapid autopsy cohort. Note that the ALK-positive case shows uniform immunoreactivity for ALK. **D,** Heatmap depicting ALK protein expression and dominant tumor morphology and molecular phenotype in 52 cases of the UW rapid autopsy cohort. Below, heatmap showing the distribution of ALK expression in different metastatic sites of the 5 positive cases.

To further evaluate ALK expression in metastatic prostate cancer, we first performed *in silico* analyses of previously published RNA-sequencing data of the University of Washington (UW) rapid autopsy cohort (Fig. 3A) and the Stand Up2 Cancer (SU2C) international dream team cohort (Fig. 3B; refs. 13, 32, 33). Given the diversity in molecular phenotypes observed in advanced metastatic prostate cancer, we divided tumors into four clinically relevant molecular subgroups based on the expression of androgen receptor (AR) signaling or neuroendocrine (NE) marker described previously (Fig. 3; ref. 13). Across all cohorts, we observed increased *ALK* mRNA levels in tumors with low AR signaling. The highest expression levels were found in AR negative neuroendocrine marker (NE)-positive tumors (AR−/NE+), consistent with NEPC (13). To study ALK protein expression in advanced metastatic prostate cancer, we performed ALK IHC on 52 cases of the UW rapid autopsy cohort. In this cohort, which was enriched for NEPCs, each case was represented with multiple metastatic sites which allowed us to determine the expression heterogeneity between and within different patients. In support of our *in silico* analyses, we found that 5 of 52 (9.6%) cases demonstrated robust ALK positivity (Fig. 3C and D). Of these ALK-positive cases, four showed neuroendocrine differentiation (AR−/NE+) and one showed absence of AR and NE marker expression (AR−/NE−; Fig. 3D; ref. 13). Notably, in cases with ALK expression, most metastatic sites tended to show expression, suggesting that *ALK* activation was shared between metastases (Fig. 3D). To determine whether genomic alterations were driving *ALK* expression in these metastases, we performed targeted sequencing on samples of case 19–045, 13–117, 13–084, and 17–017, which showed the highest *ALK* levels (Supplementary Figs. S5–S8). These studies revealed no genomic *ALK* alterations. In addition, *in silico* analyses of publicly available genomic datasets of metastatic prostate cancer (including NEPCs) showed rare, mostly nonpathogenic, single-nucleotide variants and one case with a *CTSE-ALK* fusion (Supplementary Fig. S9 and S10). Furthermore, to evaluate the presence of fusion transcripts that encompass *ALK,* we used STAR-Fusion and detected putative RNA fusions transcripts involving *ADGRG6-ALK* and *AAK1-ALK* rearrangements in two cases from the SU2C cohort (Supplementary Fig. S11; ref. 24). Neither case showed NE marker expression, and only the case harboring the *AAK1-ALK* fusion showed significantly increased *ALK* expression levels (Supplementary Figs. S11 and S12). Notably, *AAK1*, but not *ADGRG6* or *ALK*, appeared to be influenced by AR signaling (Supplementary Figs. S13 and S14). In summary, these data demonstrate that a subset of advanced prostate cancers, which are enriched for NEPC, show high level of ALK expression that is readily detectable by a validated IHC assay.

### ALK Overexpression in NEPC Is Associated with Distinct Transcriptional Programs

**FIGURE 4:**
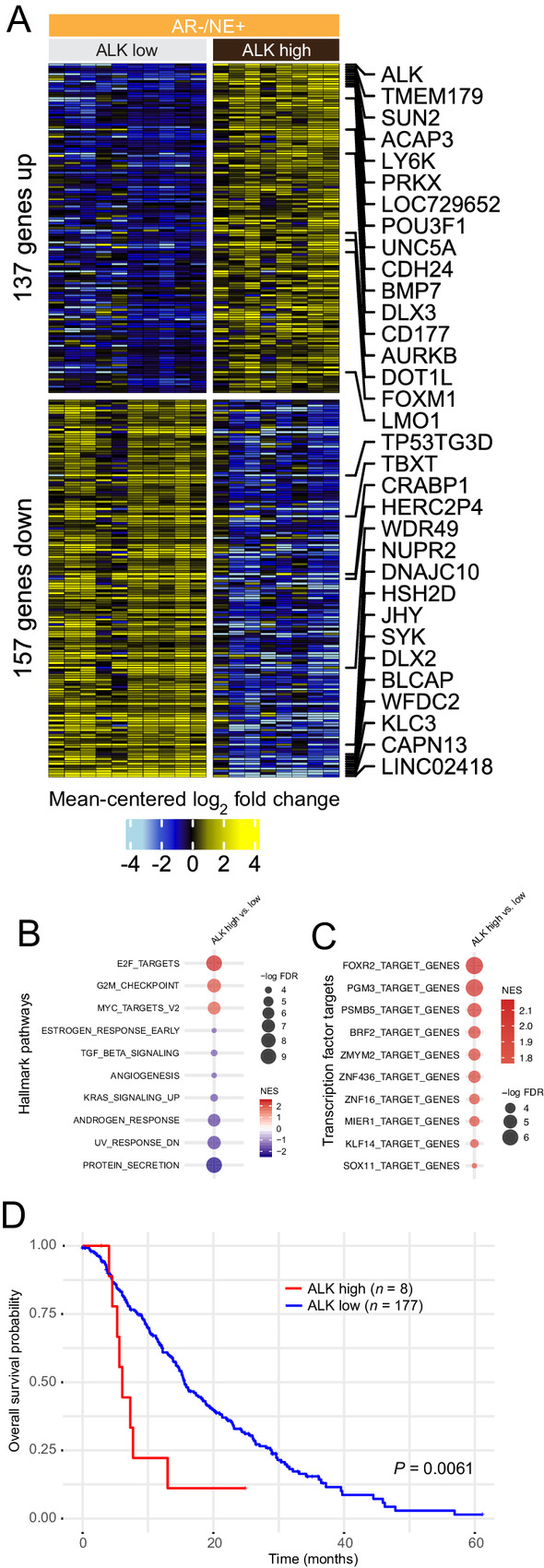
ALK overexpression is associated with distinct transcriptional changes in NEPC. **A,** Heatmap depicting expression differences between 10 tumors of the UW rapid autopsy cohort with low/no ALK expression and eight tumors with high ALK expression. Shown are mean centered log_2_ fold changes for 137 upregulated and 157 downregulated genes with FDR<0.05. **B,** Gene-set enrichment analysis for Hallmark Pathway gene sets show distinct expression differences in pathways involved in cellular proliferation and MYC signaling. **C,** Trans-cription factor signature enriched in *ALK*-overexpressing tumors. All pathways shown have enrichment FDR < 0.05. **D,** Kaplan–Meier survival plot shows shortened overall survival of patients with high ALK mRNA expression in the Stand Up to Cancer International Dream Team cohort.

Given the elevated expression of ALK in a subset of NEPC tumors, we evaluated the gene expression patterns in metastatic neuroendocrine tumor samples from the UW rapid autopsy cohort (13). To this end, we performed differential expression analyses on 10 ALK-low/negative tumors and compared them with eight tumors with high ALK expression. Using a significance cutoff of FDR<0.05, we identified 137 genes which were upregulated and 157 genes which were downregulated in NEPCs with high ALK expression (Fig. 4A). Importantly, upregulated genes included the transcription factor POU3F1 (OCT6), which has been previously implicated in nerve regeneration and is overexpressed in a subset of NEPC (34) as well as epigenetic regulators such as the histone methyltransferase DOT1L (35), the Forkhead box protein M1, FOXM1, and the G_2_–M phase gene aurora kinase B (*AURKB*). Gene-set enrichment analyses using Hallmark Pathways showed increased E2F, G2M, and MYC-targeted gene expression in tumors with elevated *ALK* levels, a signature suggestive of more proliferative and biologically more aggressive tumors (Fig. 4B). Transcription factor target analyses revealed an enrichment in FOXR2, but also PGM3 and PSMB5 gene targets in *ALK*-overexpressing tumors (Fig. 4C). Furthermore, *ALK* expression was tightly associated with the levels of neuronal transcription factors, in particular *MYCN* (Supplementary Fig. S15). However, ALK overexpression in LNCaP cells did not result in increased expression of MYCN or other neuroendocrine markers (Supplementary Fig. S16). We further investigated whether *ALK* expression would be associated with differences in clinical outcomes. To this end, we first performed Kaplan–Meier survival analyses in patient from the SU2C cohort and observed that *ALK* expression was associated with significantly shorter survival (Fig. 4D). Similar results were also observed in the UW rapid autopsy cohort (Supplementary Fig. S17A). Importantly, this association was maintained even in subset analyses restricted to NEPC tumors, suggesting that high ALK expression could be used to identify a subset of NEPCs with a particularly poor outcome (Supplementary Fig. S17B).

### ALK-Expressing NEPCs are Sensitive to ALK Inhibition

**FIGURE 5:**
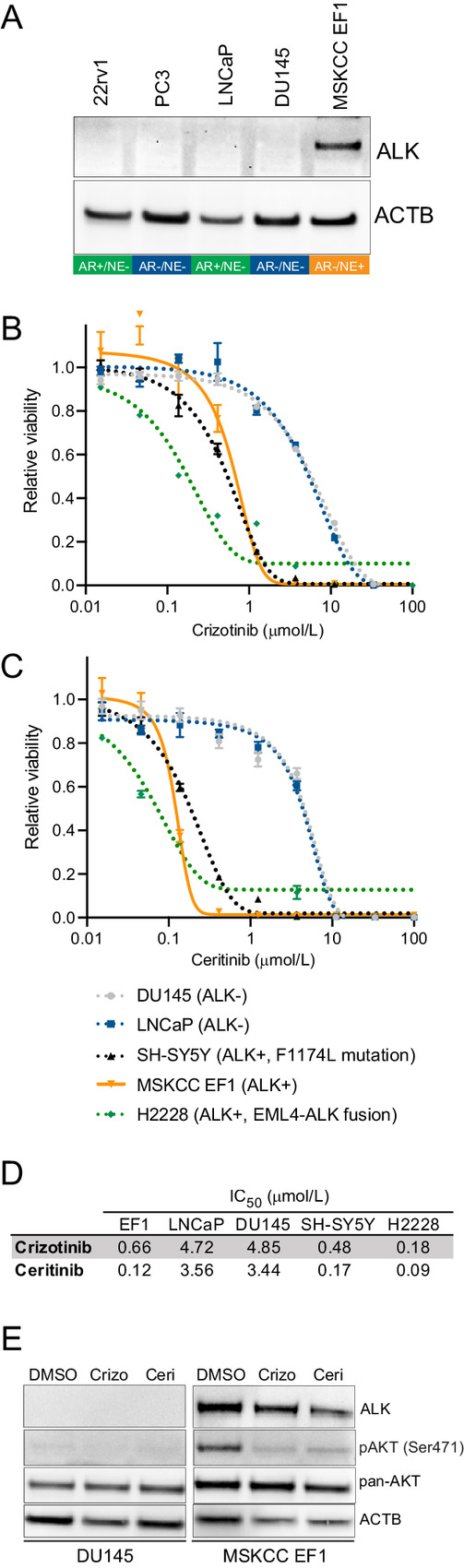
Pharmacologic inhibition of ALK in an ALK-expressing NEPC model (MSKCC EF1) results in reduced cell viability. **A,** Western blot showing ALK expression in the prostate cancer cell lines 22rv1, DU145, PC3, LNCaP, and MSKCC EF1. The molecular phenotype (AR/NE positivity) is indicated below the gel images. **B,** Dose–response curves of crizotinib in LNCaP, DU145, H2228 (EML4-ALK fusion), SH-SY5Y (F1117L ALK-activating mutation) and MSKCC EF1 cells. **C,** Dose–response curves of ceritinib in LNCaP, DU145, H2228, SH-SY5Y, and MSKCC EF1 cells. **D,** Summary table of IC_50_ values for crizotinib and ceritinib in LNCaP, DU145, H2228, SH-SY5Y, and MSKCC EF1 cells. **E,** Western blots for ALK, AKT Ser471, and pan-AKT shows increased AKT phosphorylation in MSKCC EF1 cells which is inhibited by crizotinib (crizo, 5 μmol/L) and ceritinib (ceri, 2 μmol/L) treatment for 18 hours.

Aberrant ALK activation has been shown to sensitize tumor cells to pharmacologic ALK inhibition (2). To identify prostate cancer models with high ALK expression, we assessed ALK protein levels in cell lines and patient-derived xenografts (Fig. 5A; Supplementary Fig. S18). We determined that the recently described bona fide NEPC cell line, MSKCC EF1, exhibited the highest levels of ALK protein expression and therefore represented an ideal preclinical model (17). Notably, NGS showed no genomic alterations of the *ALK* locus in MSKCC EF1 cells (Supplementary Fig. S19). To determine the response of prostate cancer cell lines to pharmacologic ALK inhibition, we performed cell viability assays across a broad concentration range for two FDA-approved ALK inhibitors (crizotinib and ceritinib) in three prostate cancer cell lines as well as the lung cancer cell line H2228, which harbors an *EML4-ALK* fusion and the neuroblastoma cell line SH-SY5Y which has an *ALK*-activating mutation (6). Both H2228 and SH-SY5Y have been previously shown to be sensitive to ALK inhibition (36, 37). We observed that MSKCC EF1 was substantially more sensitive than any other prostate cancer cell line (Fig. 5B and C) and showed comparable IC_50_ values to H2228 and SH-SY5Y (Fig. 5D). To further query the molecular consequences of ALK inhibition, we performed Western blot analyses in ALK-positive and ALK-negative cell lines for AKT activation. ALK-positive MSKCC EF1 cells showed a high baseline level of AKT activation as determined by phosphorylation at Ser473 of AKT which was abrogated after a 18-hour treatment with crizotinib or ceritinib (Fig. 5E). This finding is consistent with AKT signaling changes in ALK-rearranged lung cancers (8). Collectively, these experiments provide evidence that ALK-overexpressing prostate cancers can be targeted with small-molecule ALK inhibitors and suggest that ALK inhibitors might represent a potential therapeutic option for advanced prostate cancers with high ALK expression.

## Discussion

Context- and cell lineage–dependent genomic alterations or overexpression of receptor tyrosine kinases result in signaling dependencies in cancer cells, which can be exploited therapeutically. ALK is one of the tyrosine kinase receptors that has been credentialled and clinically validated as a therapeutic target in solid tumors (38). Although first discovered as a rearranged gene in anaplastic lymphoma, *ALK* gene fusions have been documented in numerous tumor types (2, 4). ALK inhibitors are established as first-line therapies for lung cancers with ALK alterations (10). Through the widespread use of NGS, an increasing number of tumor types has been shown to harbor *ALK* alterations, suggesting that ALK-directed therapies could be of used outside of its current established indications (4, 5).

Although prostate cancer has not been thought of as an ALK-driven tumor, anecdotal evidence suggests that ALK genomic and expression alterations can be observed in prostate cancer (16, 39). Here we assessed the spectrum of ALK alterations in localized and advanced metastatic prostate cancer. To this end, we used a validated IHC assay to detect ALK protein expression. Prior studies have shown that ALK IHC is a robust readout for assessing ALK expression and indirect detection of *ALK* rearrangements (28, 40). In our cohort of 372 localized prostate cancers, we identified a single case with strong and uniform ALK expression. This lesion had a cribriform architecture, which is the most common morphology seen in ALK-positive lung adenocarcinomas, suggesting at least some lineage-independent overlap of histomorphologic features in cases with *ALK* alterations (41).

Genomic analyses in this case revealed a structural genomic alteration resulting in the fusion of the androgen-regulated gene *SLC45A3* with *ALK*. The *SLC45A3* gene locus is frequently involved in genomic alterations in prostate cancer. In around 6% of *ERG* rearranged prostate cancers, *SLCA45A3* represents the 5′ fusion partner of *ERG* (29). Notably, whereas most *ALK* rearrangements in lung, breast, and colon cancers mostly involve intron 19, the rearrangements in this case occurred in intron 15, resulting in an overexpressed truncated ALK protein encompassing exon 16–29 (5, 42). Although it is unclear whether the inclusion of exon 16–19 in the fusion transcript has any biological significance, this finding has relevance for the design of targeted sequencing panels. To capture a broader spectrum of *ALK* rearrangements, inclusion of additional intronic and exonic sequences upstream of the canonically rearranged exon 19 might increase the sensitivity to detect *ALK* alterations (25).

Functionally, overexpression of the truncated ALK16–29 transcript in benign prostate epithelial cells resulted in dramatically increased cell proliferation that was greatly inhibited by the addition of the ALK catalytic inhibitor crizotinib. On the basis of this result, it is likely that rare, oncogenic ALK alterations represent oncogenic drivers in prostate cancer that can be targeted by available ALK inhibitors.

In our expanded survey of advanced metastatic prostate cancers, we observed increased ALK expression in a subset of prostate cancer metastases. Importantly, in *in silico* analyses, high-level ALK mRNA expression was strongly enriched in tumors with low/no AR expression and evidence of neuroendocrine differentiation. This finding is reminiscent of neuroblastoma, a tumor with primitive neuroendocrine differentiation which is known to show increased ALK expression and ALK genomic alterations in approximately 10% of cases (43). This suggests that in the context of neuronal/neuroendocrine lineage differentiation, ALK expression could be a particularly relevant contributor to tumor progression.

ALK expression in metastatic prostate cancers was associated with distinct transcriptional changes. Notably, gene-set enrichment analyses suggested an increased representation of genes involved in cell proliferation and MYC signaling, which have been associated with more aggressive phenotypes. Indeed, in neuroblastoma and in other models, ALK activation has been shown to cooperate with MYCN but also regulate MYCC expression (44–47). In addition, transcription factor target analyses revealed the strongest enrichment for FOXR2 in cases with high ALK expression. It is important to note that FOXR2 activity is associated with poor prognosis in neuroblastoma where it was shown to stabilize MYCN protein (48). Furthermore, *MYCN* mRNA levels were correlated with *ALK* expression, supporting the notion that ALK and MYCN cooperate in cancer progression (46). ALK overexpression alone might therefore be insufficient to induce large-scale transcriptional changes and activation of other transcription factors are required to induce the gene signatures associated with ALK. Nevertheless, our outcome analyses suggest that ALK expression identifies a group of aggressive tumors with distinct biological behavior.

Our findings of high ALK expression in a subset of NEPC patients have potential important therapeutic implications. *In vitro* data presented here demonstrate that the ALK-overexpressing NEPC cell line MSKCC EF1 is sensitive to the ALK inhibitors crizotinib and ceritinib and show IC_50_ values similar to the ALK-rearranged lung cancer (H2228) or ALK-mutated neuroblastoma (SH-SY5Y) cell lines. In support of this, a recent case report demonstrated efficacy of the ALK inhibitor alectinib in a *de novo* prostatic small-cell carcinoma harboring a p.F1174C *ALK* mutation (16). Together, these studies support the notion that prostate cancers with increased ALK expression or putative driver genomic alterations might be sensitive to pharmacologic ALK inhibition.

In addition to ALK small-molecule inhibitors, the armamentarium of approaches to target ALK-overexpressing tumors is expanding. In a proof-of-concept study, a novel ALK targeting antibody–drug conjugate showed efficacy in preclinical neuroblastoma models (49). Importantly, this effect was independent of genomic ALK alterations making it particularly attractive for tumor types with high ALK protein expression in the absence of ALK-activating mutations or rearrangements, such as NEPC. Given the highly favorable expression pattern of ALK with no/limited detectable protein expression in benign tissues, antibody–drug conjugate or cell-based therapies exploiting the cancer-specific expression of ALK could be attractive options for prostate cancer (49, 50).

In summary, the data presented here demonstrate that a subset of prostate cancers show potentially actionable alterations of the tyrosine receptor kinase ALK. This warrants further clinical testing of ALK-targeting agents such as small-molecule inhibitors and antibody–drug conjugates in selected patients with proven ALK expression/genomic alterations.

## Supplementary Material

Supplementary Figures 1-19, Tables 1-2 Supplementary Figures 1-19, Tables 1-2
