# Supplementary material for: Comprehensive Assessment of Anaplastic Lymphoma Kinase in Localized and Metastatic Prostate Cancer Reveals Targetable Alterations
Source: Cancer Res Commun. 2022 May 2;2(5):277–85. doi: 10.1158/2767-9764.CRC-21-0156 (PMC9635400; doi:10.1158/2767-9764.CRC-21-0156)
Supplement: Supplementary Figures 1-19, Tables 1-2 [file crc-21-0156-s01.pdf]

SUPPPLEMENTARY DATA

Comprehensive assessment of anaplastic lymphoma kinase in localized and metastatic prostate cancer reveals targetable alterations.

Radhika A. Patel, Ilsa Coleman, Martine P. Roudier, Eric Q. Konnick, Brian Hanratty, Ruth Dumpit, Jared M. Lucas, Lisa S. Ang, Jin-Yih Low, Maria S. Tretiakova, Gavin Ha, John K. Lee, Lawrence D. True, Angelo M. De Marzo, Peter S. Nelson, Colm Morrissey, Colin C. Pritchard, Michael C. Haffner

Supplementary Figure 1. *Patel et al.*

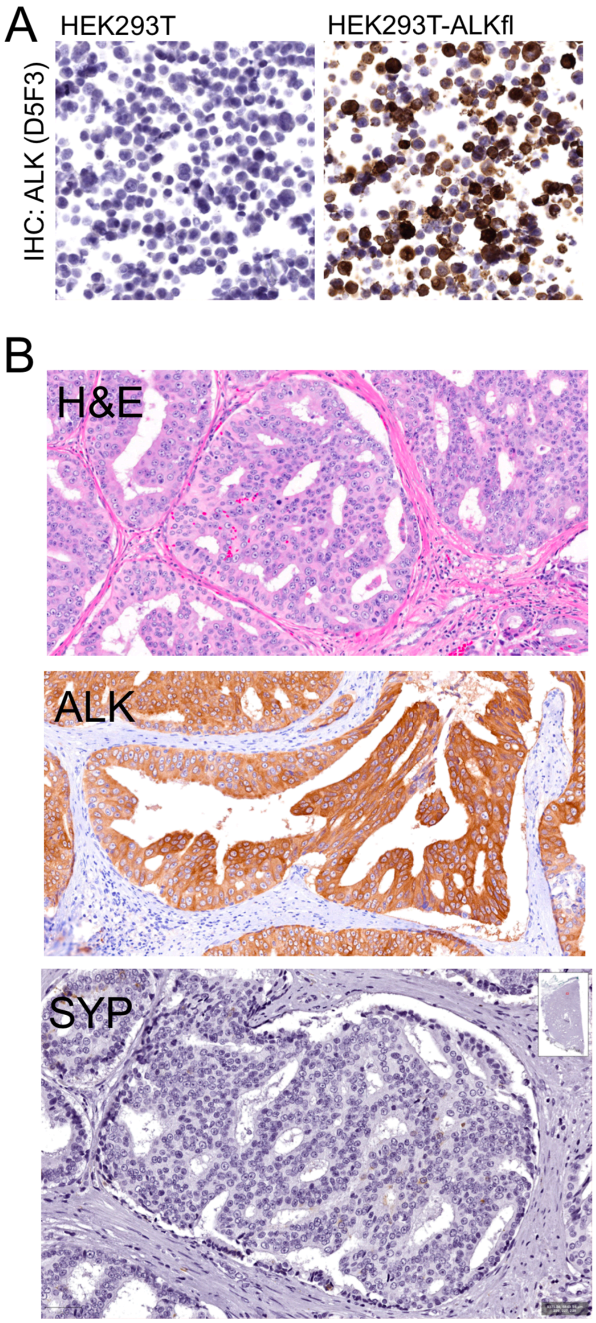

SUPPLEMENTARY FIGURE 1. A. HEK293T cells (known to be ALK negative) and HEK293T expression full length (fl) ALK serve as genetically defined negative and positive controls for the ALK immunohistochemical assay using ALK specific antibody clone D5F3. B. Representative H&E, ALK IHC and synaptophysin IHC images demonstrate that the ALK rearranged tumor shows cribriform growth, robust cytoplasmic positivity for ALK and absence of synaptophysin expression.

Supplementary Figure 2. *Patel et al.*

A

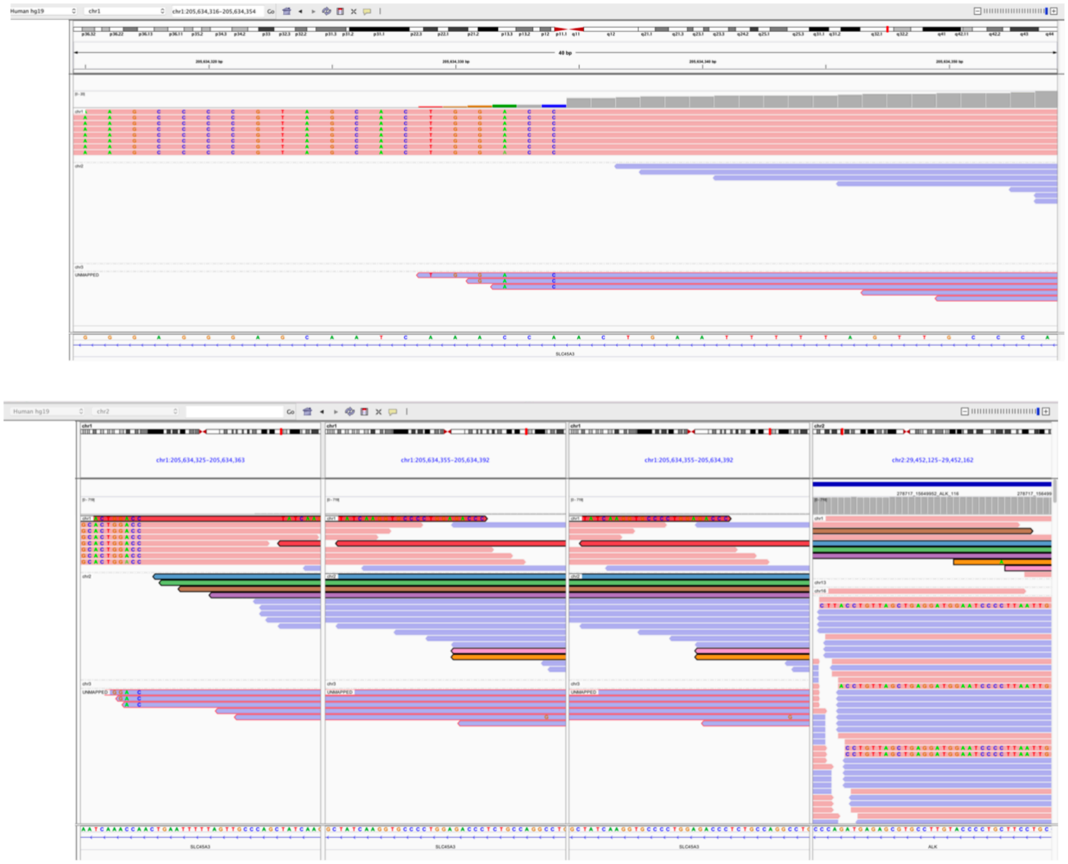

B

Genomic altereations in ALK positive case:
ALK-SLC45A3 fusion (approximate hg19 genomic coordinates are chr2:29452318, ALK intron 15; and chr1:205634335, SLC45A3 intron 1),
CHEK2 p.V198Ffs*7
AKT1 (p.E17K, NM_001014431.1:c.49G>A, 20% variant
fraction)
BRCA2 copy loss (cannot determine if mono-allelic or bi-allelic),
FOXA1 (1.p.R265G, NM_004496.3:c.793C>G, 6% variant fraction, 2. p.T445_E451del, NM_004496.3:c.1333_1353del 7% fraction, and 3. 57bp del in exon 2
NEGATIVE for microsatellite instability, with
Variants of uncertain significance are noted in
MTOR (p.R2270W, NM_004958.3:c.6808C>T, 28% fraction, and p.N763S
NM_004958.3:c.2288A>G, 27% fraction)
POLE (p.Y224*, NM_006231.2:c.672C>G, 19% fraction)
KMT2C (p.E3199*,NM_170606.3:c.9595G>T, 36% fraction)
ALK (p.G1450Pfs*29,NM_004304.4:c.4343_4347dup, 18% fraction)
SLX4 (p.R1666Q, NM_032444:c.4997G>A, 20% fraction).

SUPPLEMENTARY FIGURE 2. A. IGV screenshot demonstrating SLC45A3-ALK rearrangement. B Additional genomic findings in this case.

Supplementary Figure 3. *Patel et al.*

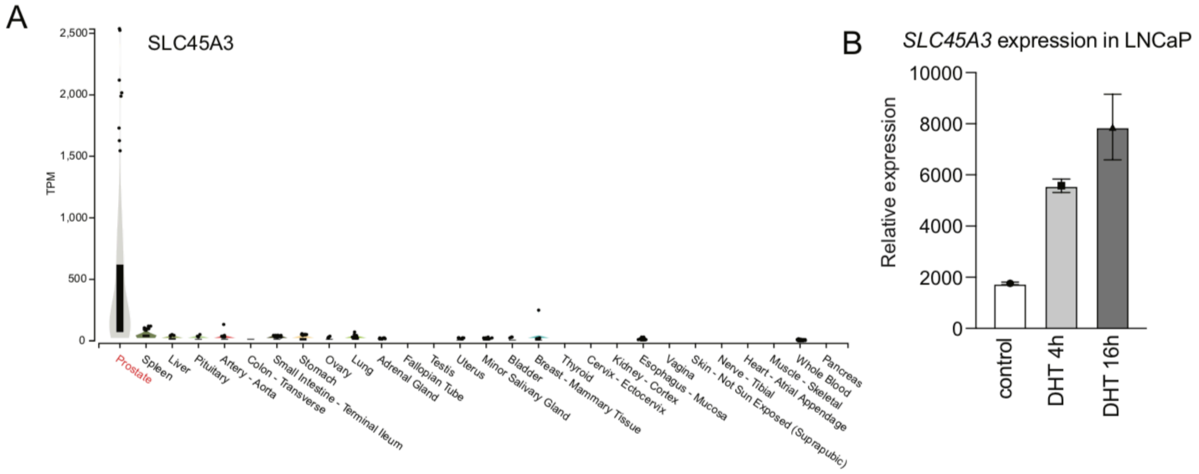

SUPPLEMENTARY FIGURE 3. A. Expression analysis using the GTEx database show that high level *SLC45A3* expression is restricted to the prostate. B. *SLC45A3* is an androgen regulated gene.

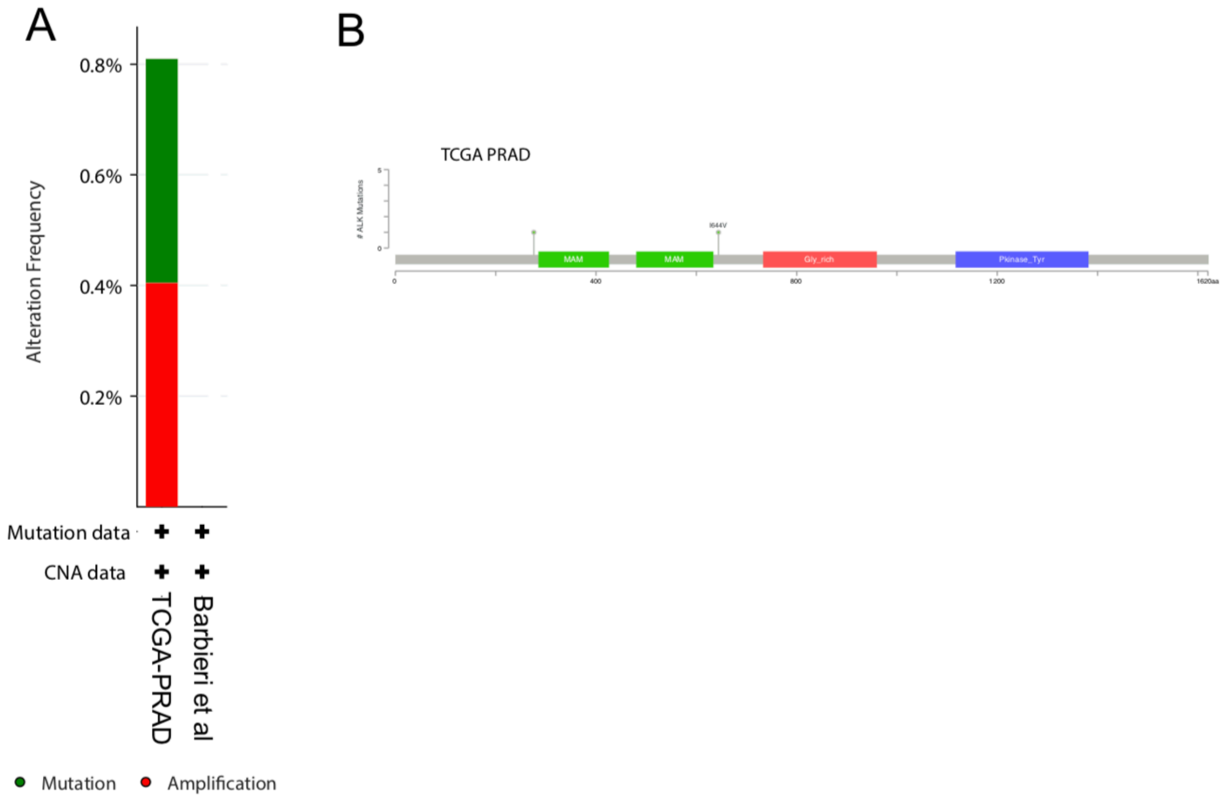

SUPPLEMENTARY FIGURE 4. A. Genomic *ALK* alteration frequencies in localized prostate cancers from the TCGA-PRAD cohort (N=494) (1) and Barbieri et al. (N=112) (2). B. Missense mutation in ALK in the TCGA-PRAD cohort. Note that the detected missense mutations are likely non-pathogenic and non-recurrent outside of the kinase domain. Data and figures were extracted from the cBio Cancer Genomics Portal (3)

Supplementary Figure 5. *Patel et al.*

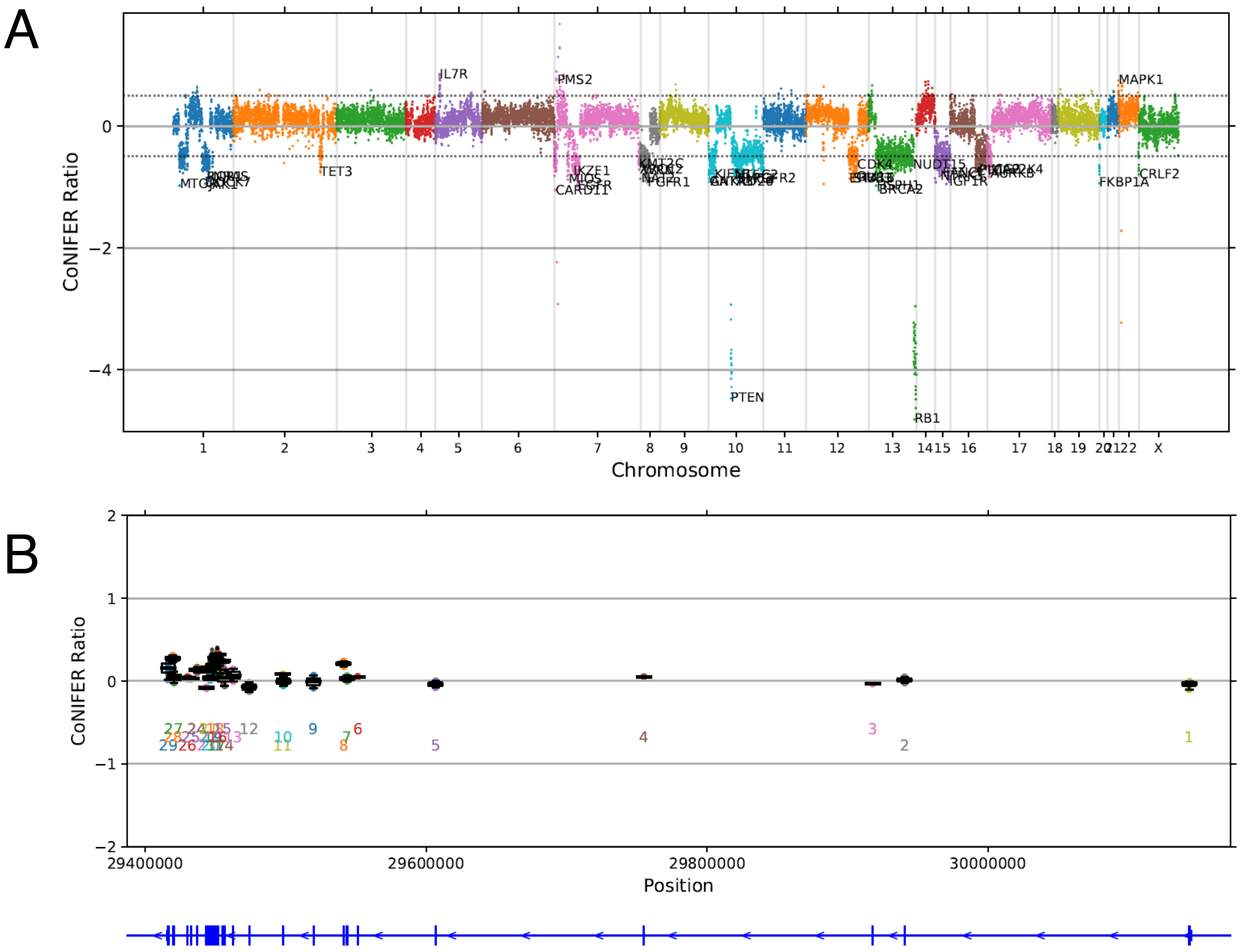

ALK

SUPPLEMENTARY FIGURE 5. A. Chromosome wide copy-number analysis and B. normalized copy-number of the *ALK* locus show no evidence for ALK genomic alterations in case 19-045.

Supplementary Figure 6. *Patel et al.*

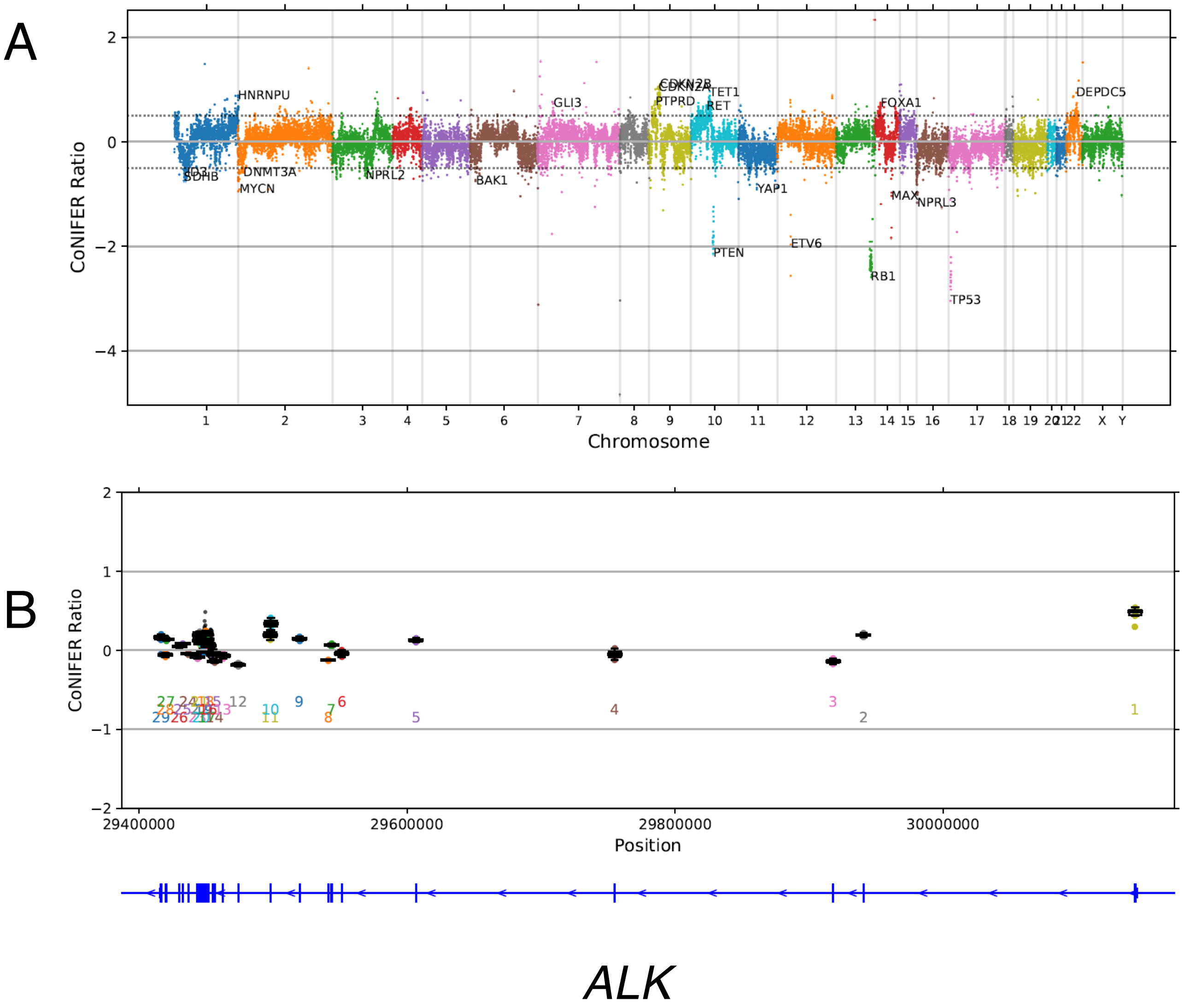

SUPPLEMENTARY FIGURE 6. A. Chromosome wide copy-number analysis and B. normalized copy-number of the *ALK* locus show no evidence for ALK genomic alterations in case 13-117.

Supplementary Figure 7. *Patel et al.*

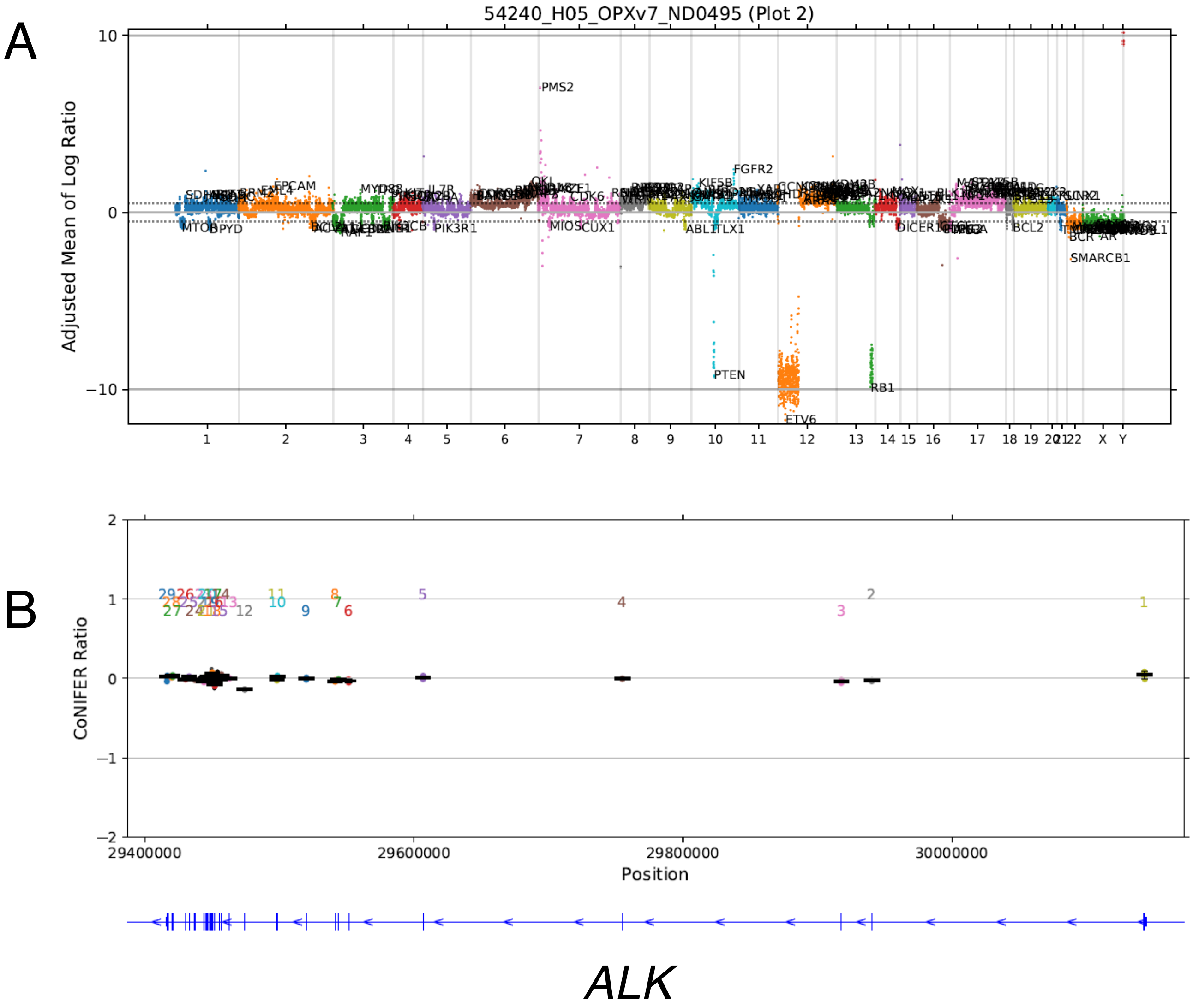

SUPPLEMENTARY FIGURE 7. A. Chromosome wide copy-number analysis and B. normalized copy-number of the *ALK* locus show no evidence for ALK genomic alterations in case 13-084.

Supplementary Figure 8. *Patel et al.*

A

B

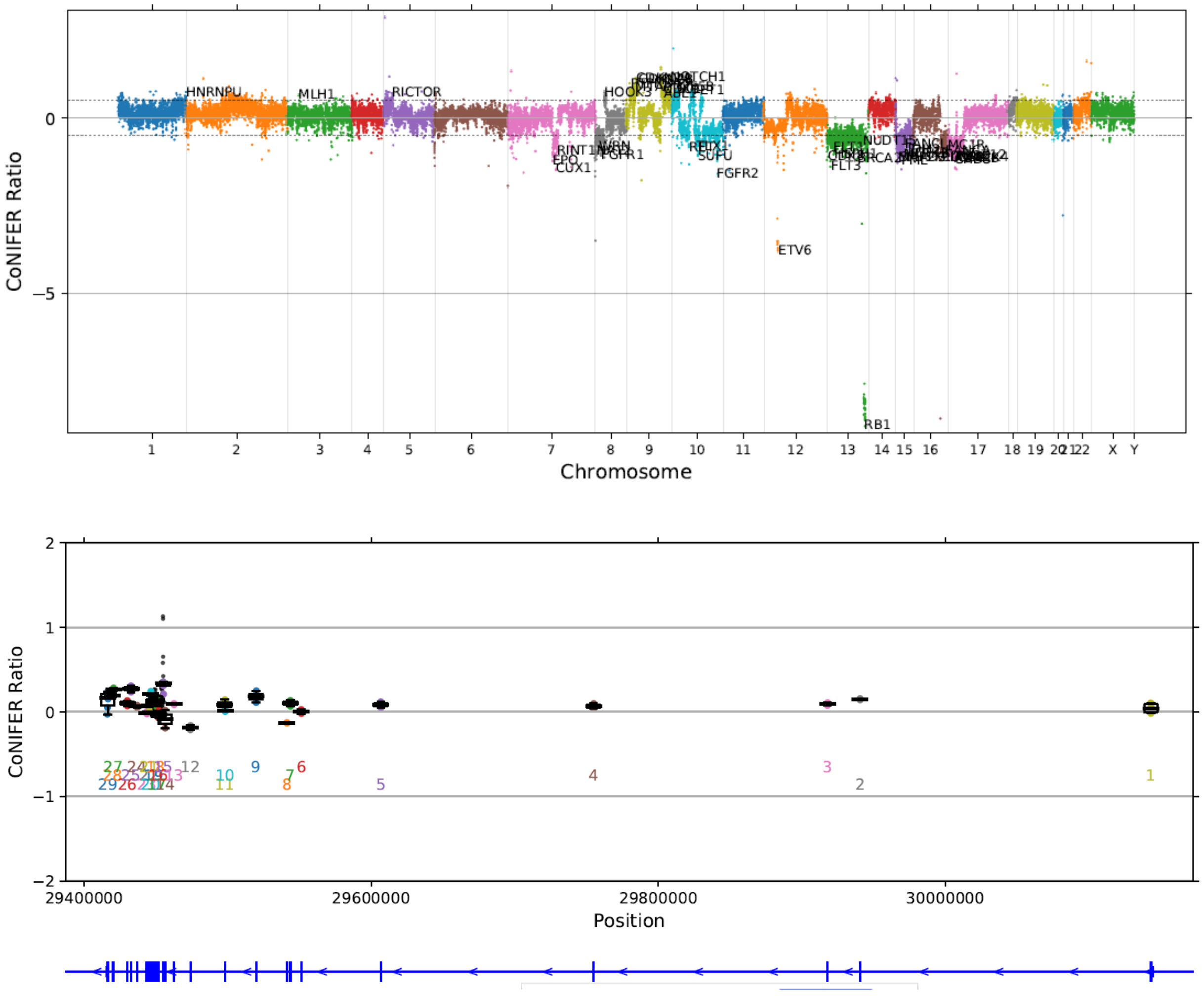

ALK

SUPPLEMENTARY FIGURE 8. A. Chromosome wide copy-number analysis and B. normalized copy-number of the *ALK* locus show no evidence for ALK genomic alterations in case 17-017.

A

P-0001768-T01-IM3	*R1275Q*	Missense
P-0024679-T01-IM6	*CTSE-ALK fusion*	Fusion
P-0045746-T01-IM6	*C1156F*	Missense
P-0042957-T01-IM6	*X472_splice*	Splice
P-0031855-T01-IM6	*G822Vfs*9*	FS del
P-0034845-T01-IM6	*G822Vfs*9*	FS del
P-0002984-T01-IM3	*G925**	Nonsense
P-0021310-T01-IM6	*D305Efs*30*	FS del
P-0013115-T01-IM5	*X786_splice*	Splice
P-0035133-T01-IM6	*W915Gfs*24*	FS del
P-0039071-T01-IM6	*N369Sfs*2*	FS del
P-0002984-T01-IM3	*G925**	Nonsense
P-0021310-T01-IM6	*D305Efs*30*	FS del
P-0013115-T01-IM5	*X786_splice*	Splice
P-0043336-T01-IM6	*ALK-intragenic*	Fusion

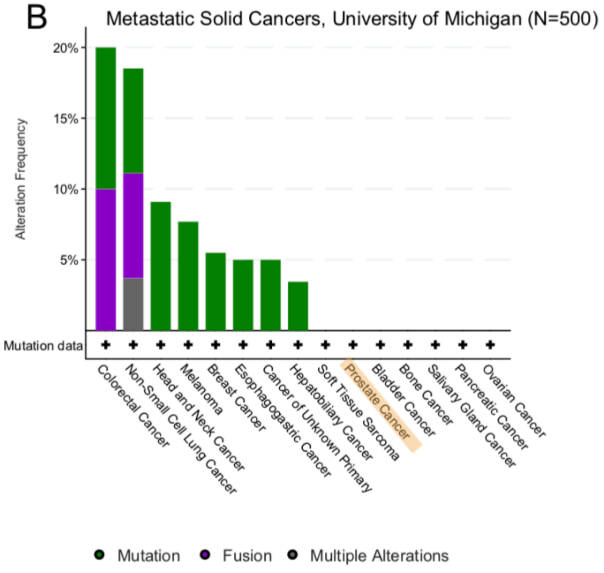

SUPPLEMENTARY FIGURE 9. A. *ALK* genomic alterations in 3534 samples of the MSK-IMPACT targeted sequencing panel shows rare cases with ALK genomic changes (4). B. *ALK* genomic alterations across 500 metastatic solid tumors (5). Data and figures were extracted from the cBio Cancer Genomics Portal (3).

Supplementary Figure 10. *Patel et al.*

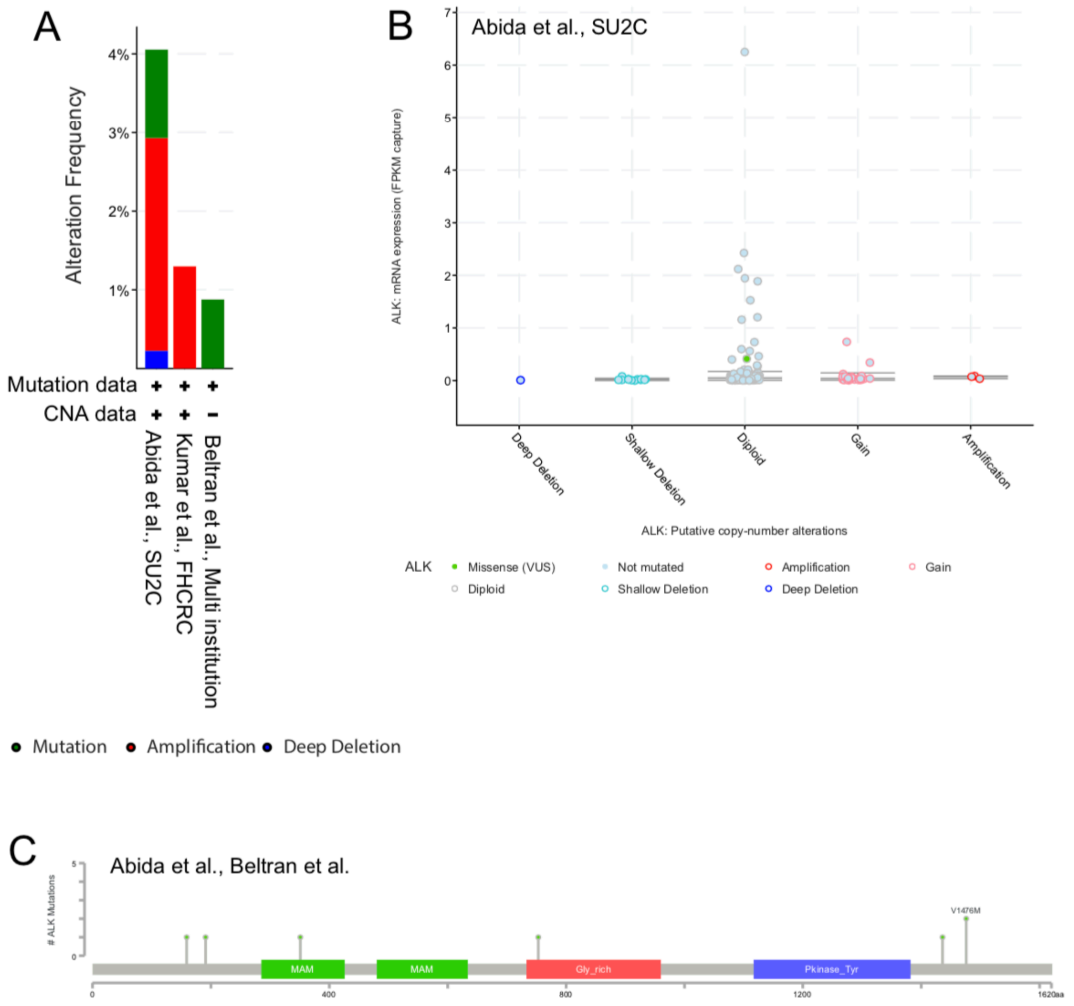

SUPPLEMENTARY FIGURE 10. A. *ALK* mutation and copy-number alterations in metastatic prostate cancers from the SU2C/Abida et al (N=444) (6), the Fred Hutchinson Cancer Center/Kumar et al. (N=176) (7) and the Beltran et al. (N=114) (8) cohorts. B. Association between *ALK* copy-number and *ALK* mRNA expression in the SU2C/Abida et al cohort. Note that *ALK* amplification was not associated with increased ALK mRNA expression. C. Location of ALK mutations from SU2C/Abida et al and Beltran et al.. All detected missense mutations are in non-hotspot locations and are outside of the kinase domain. Data and figures were extracted from the cBio Cancer Genomics Portal (3).

Supplementary Figure 11. *Patel et al.*

A

X.FusionName	JunctionReadCount	SpanningFragCount	est_J	est_S	LeftGene	LeftBreakpoint	RightGene	RightBreakpoint	FFPM	annots	cBioPortal_ID	Molecular Phenotype
ADGRG6--ALK	10	0	10	0	ADGRG6	chr6:142370793:+	ALK	chr2:29222607:-	0.2573	[INTERCHROMOSOMAL[chr6--chr2]]	MO_1541_	AR low, NE negative
AAK1--ALK	67	0	67	0	AAK1	chr2:69642878:-	ALK	chr2:29717697:-	1.3979	[INTRACHROMOSOMAL[chr2:39.54Mb]]	SC_9203_	AR positive, NE negative

B

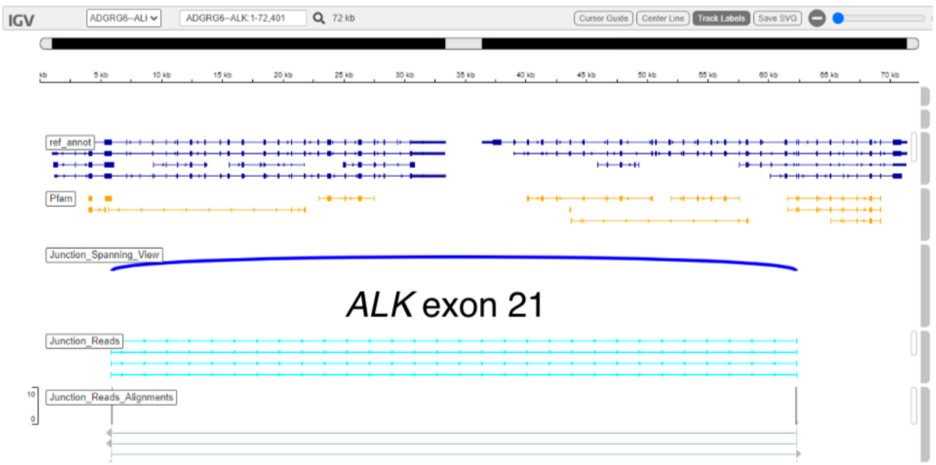

C

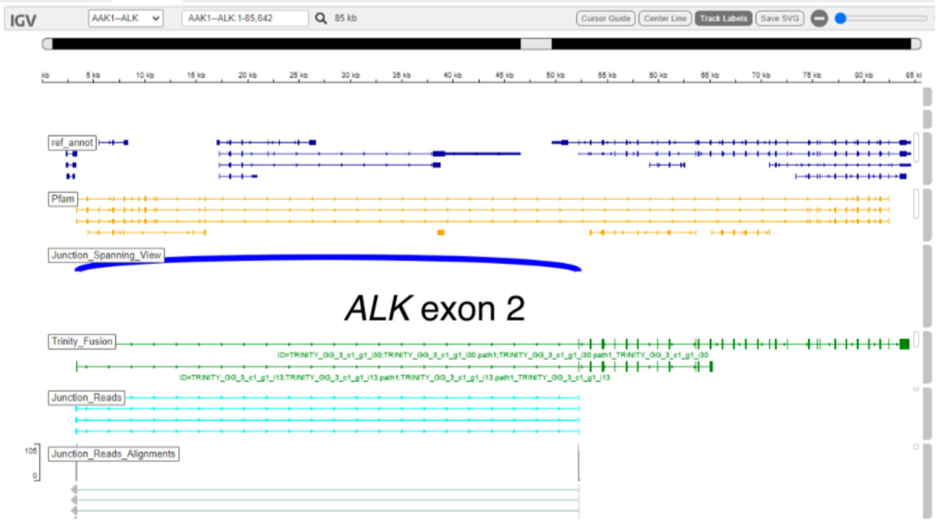

SUPPLEMENTARY FIGURE 11. A. ALK fusion transcripts in SU2C**.** IGV screen shot demonstrating supporting reads for *ADGRG6-ALK* (B) and *AAK1-ALK* (C) rearrangements in 2 cases from the SU2C cohort.

Supplementary Figure 12. *Patel et al.*

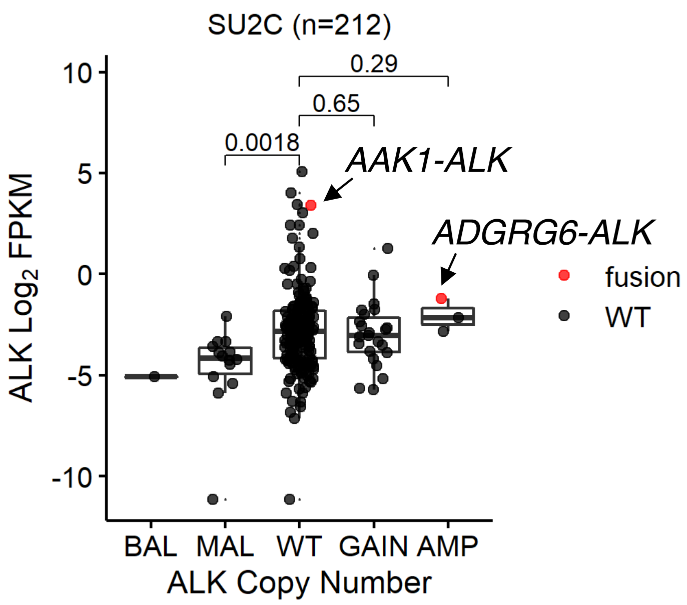

SUPPLEMENTARY FIGURE 12. Association between *ALK* gene locus copy-number and *ALK* expression in the capture RNA-seq cohort of the SU2C International dream team study. Note that there is no statistically significant difference in ALK expression between WT and *ALK* copy-number gained cases. The red dots indicate 2 cases with *ALK* rearrangements based on star-fusion analysis.

Supplementary Figure 13. *Patel et al.*

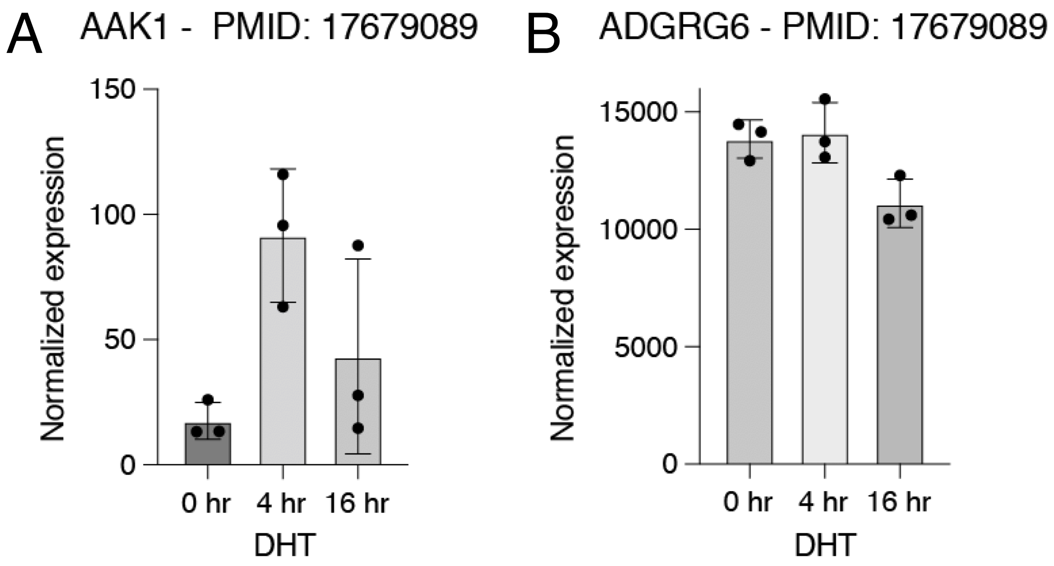

SUPPLEMENTARY FIGURE 13. Analyses of publicly available gene expression studies in androgen deprived (0 hr) and dihydrotestosterone (DHT, 4 hr, 16 hr) treated LNCaP cells reveal increased expression of AAK1 (A), but no significant changes in ADGRG6 expression (B).

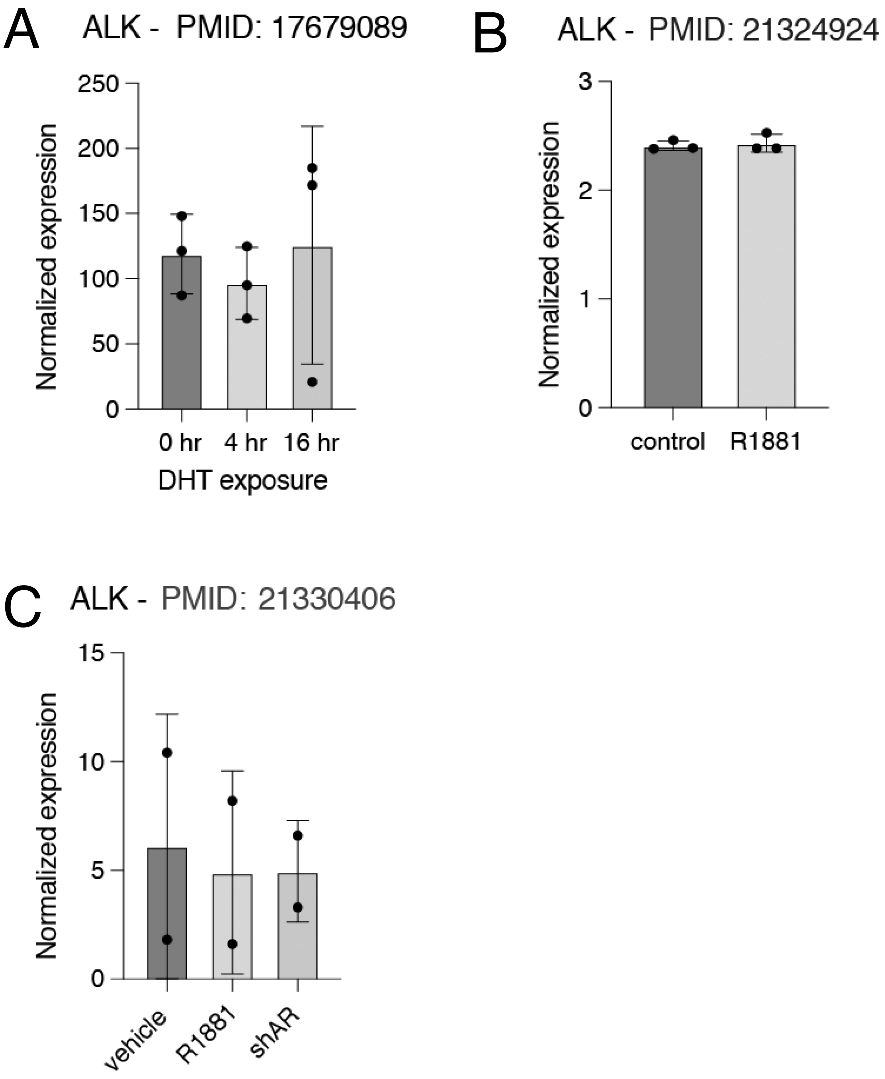

SUPPLEMENTARY FIGURE 14. Analyses of 3 independent publicly available gene expression studies (A, B, C) show no evidence for AR/androgen mediated regulation of ALK in prostate cancer cells.

Supplementary Figure 15. *Patel et al.*

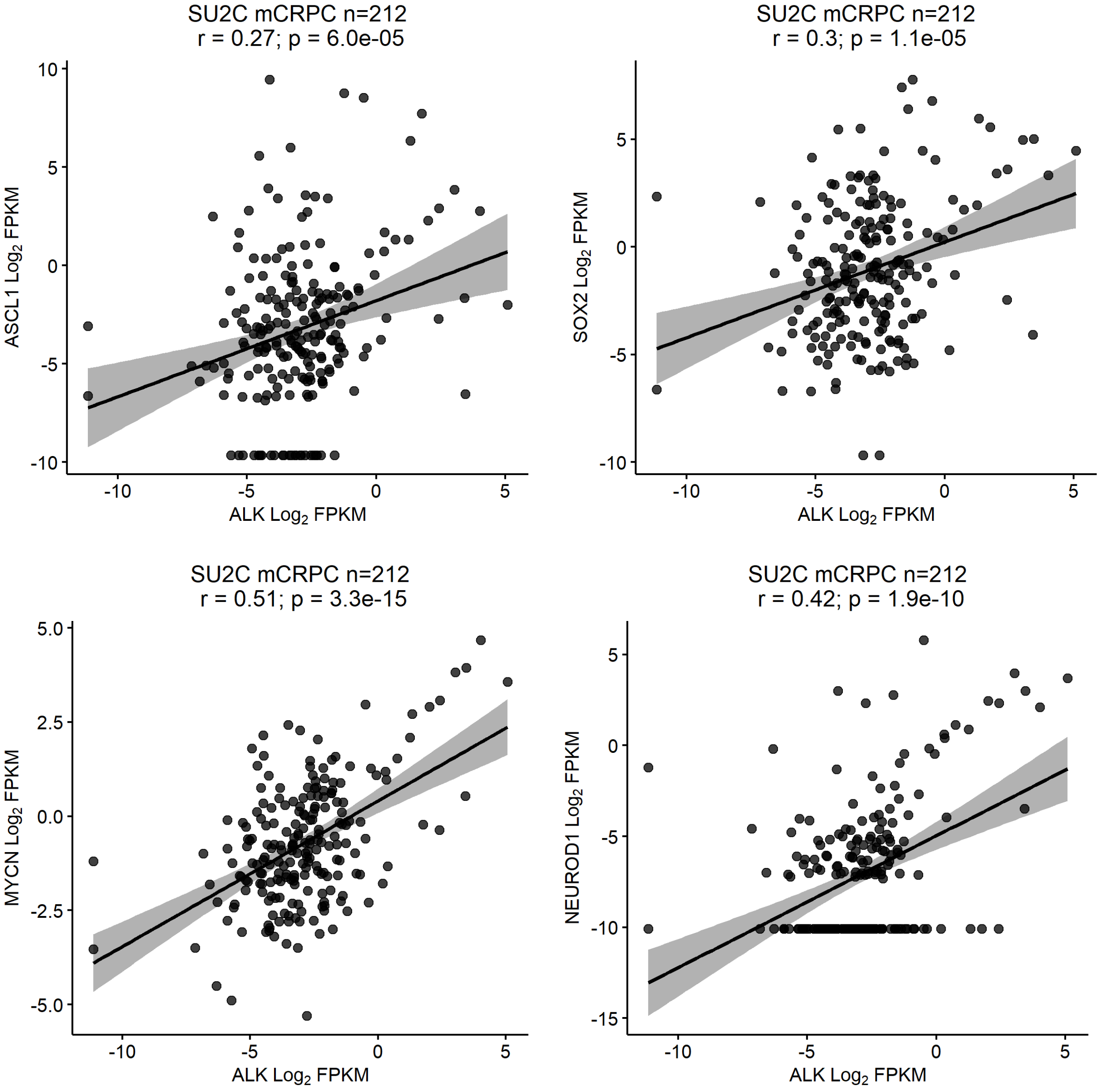

SUPPLEMENTARY FIGURE 15. Gene transcript correlation matrices based on analyses of 212 cases from the SU2C cohort show correlation between ALK and ASCL1, SOX2, NEUROD1 and MYCN mRNA expression.

Supplementary Figure 16. *Patel et al.*

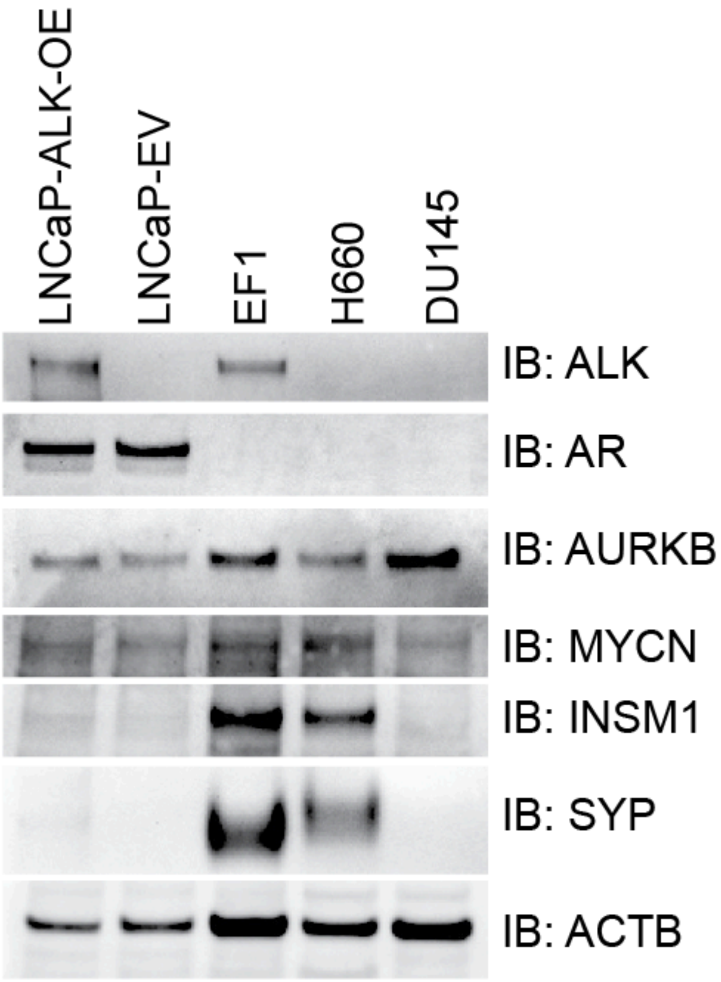

SUPPLEMENTARY FIGURE 16. Western blot showing protein expression of ALK, AR, AURKB, MYCN, INSM1, SYP and ACTB in ALK overexpressing LNCaP (LNCaP-ALK-OE) empty vector control LNCaP (LNCaP-EV, MSKCC EF1, NCI-H660 and DU145 cells.

Supplementary Figure 17. *Patel et al.*

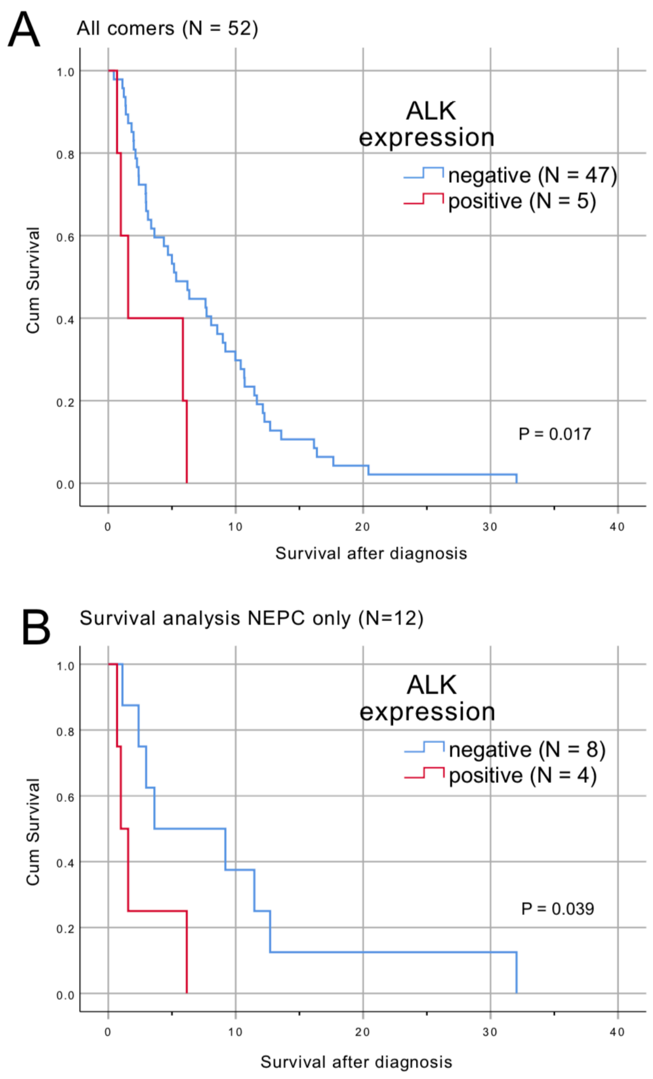

SUPPLEMENTARY FIGURE 17. Kaplan Meier graphs showing the time from initial diagnosis to death in patients from the University of Washington Rapid Autopsy cohort stratified by the presence or absence of ALK protein expression at the time of autopsy. A. Analysis of all 52 cases investigated in this study, independent of molecular phenotypes) shows shorter time to death in patients with ALK expression (mean survival 7.3 vs. 3.0 years, Log Rank P = 0.017). B. In subset analysis of 12 cases with predominant neuroendocrine prostate cancer ALK expression maintains its prognostic value (mean survival 9.4 vs. 2.3 years, Log Rank P = 0.039), suggesting that ALK expression identified a group of more aggressive variants of NEPC.

Supplementary Figure 18. *Patel et al.*

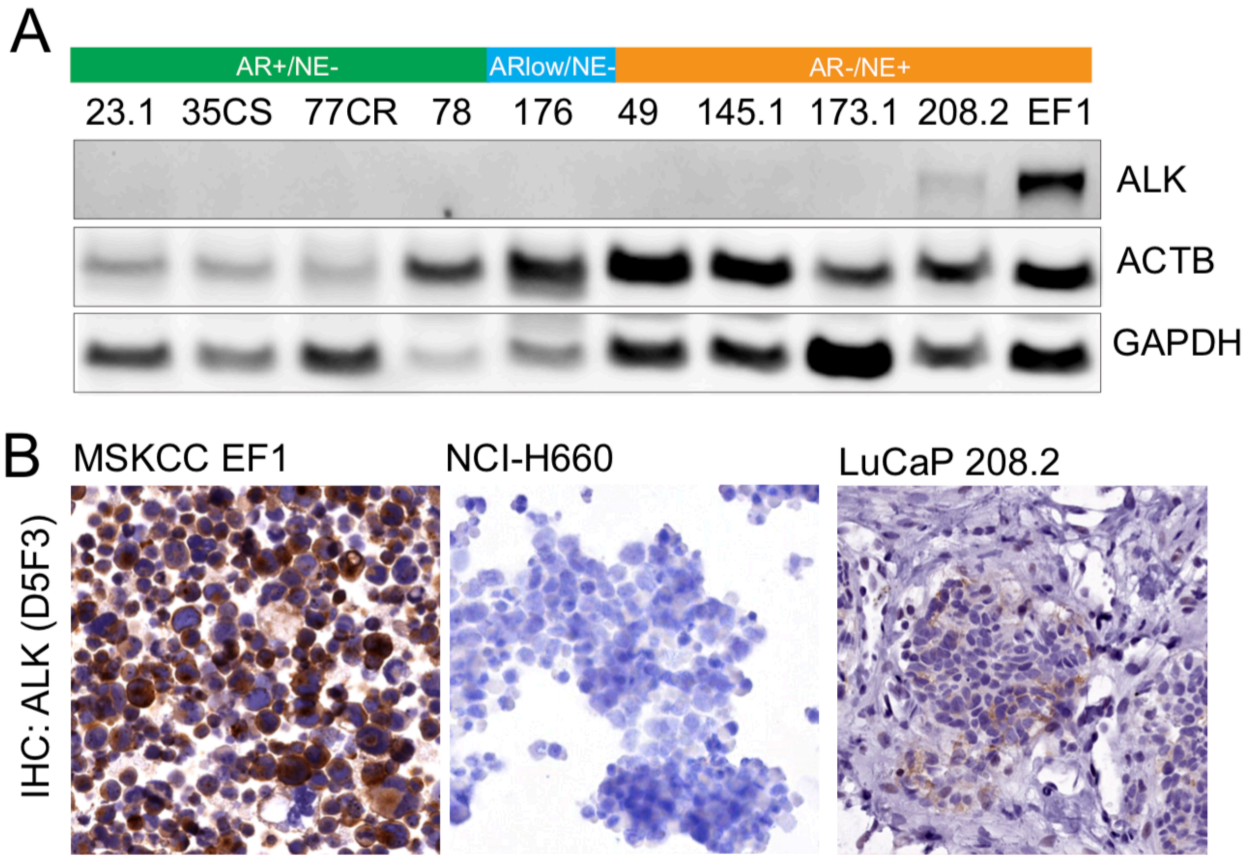

SUPPLEMENTARY FIGURE 18. A. Western blot showing ALK protein expression in LuCaP patient derived xenografts (PDX) and the cell line MSKCC EF1. Note that only MSKCC EF1 shows high level ALK expression, although a faint ALK specific band is also detected in the NEPC PDX line LuCaP 208.2. B. In situ immunohistochemical labeling shows uniform strong staining for ALK in MSKCC EF1 and scattered weak expression in LuCaP 208. The NEPC cell line NCI-H660 shows no staining for ALK.

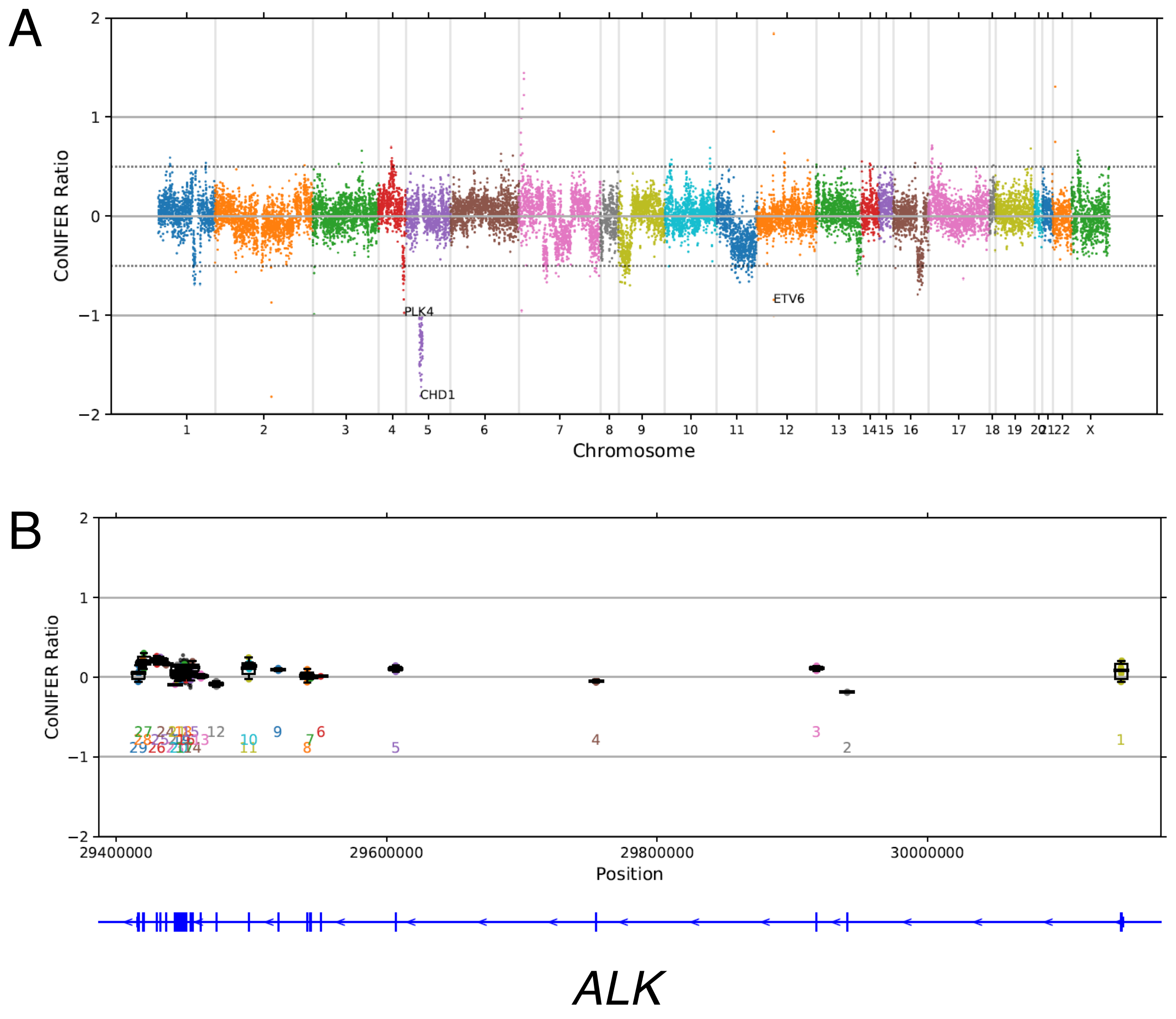

SUPPLEMENTARY FIGURE 19. A. Chromosome wide copy-number analysis and B. normalized copy-number of the *ALK* locus show no evidence for ALK genomic alterations in the MSKCC EF1 cell line.

Supplementary Table 1. Overview of clinicopathological characteristics of primary tumors tested for ALK expression

Total N = 372

		N	%
**Grade group**		N	%
	1	13	*3.5*
	2	137	*36.8*
	3	125	*33.6*
	4	60	*16.1*
	5	37	*9.9*
**Stage**	T2	171	*46.0*
	T3A	143	*38.4*
	T3B	56	*15.1*
	TX	2	*0.5*
**Lymph nodes**	N0	321	*86.3*
	N1	46	*12.4*
	NX	5	*1.3*

Supplementary Table 2. Overview of clinicopathological characteristics of metastatic prostate cancer cohort.

Total N = 52	
**Demographics data**	
Age at initial diagnosis (median [IQR])	60.61 [53.92, 66.33]
PSA at diagnosis (median [IQR])	19.50 [7.55, 111.25]
Age at death (median [IQR])	68.63 [61.91, 74.43]
PSA at death (median [IQR])	90.63 [8.69, 1007.51]
**Treatment history**	
ADT	51 (98)
Abiraterone acetate	33 (65)
Enzalutamide	28 (55)

Supplementary References. *Patel et al.*

1. Cancer Genome Atlas Research Network. The Molecular Taxonomy of Primary Prostate Cancer. Cell. 2015;163:1011–25.

2. Barbieri CE, Baca SC, Lawrence MS, Demichelis F, Blattner M, Theurillat J-P, et al. Exome sequencing identifies recurrent SPOP, FOXA1 and MED12 mutations in prostate cancer. Nat Genet. Nature Publishing Group; 2012;44:685–9.

3. Gao J, Aksoy BA, Dogrusoz U, Dresdner G, Gross B, Sumer SO, et al. Integrative analysis of complex cancer genomics and clinical profiles using the cBioPortal. Sci Signal. 2013;6:pl1–pl1.

4. Zehir A, Benayed R, Shah RH, Syed A, Middha S, Kim HR, et al. Mutational landscape of metastatic cancer revealed from prospective clinical sequencing of 10,000 patients. Nat Med. Nature Publishing Group; 2017;23:703–13.

5. Robinson DR, Wu Y-M, Lonigro RJ, Vats P, Cobain E, Everett J, et al. Integrative clinical genomics of metastatic cancer. Nature. Nature Publishing Group; 2017;548:297–303.

6. Abida W, Cyrta J, Heller G, Prandi D, Armenia J, Coleman I, et al. Genomic correlates of clinical outcome in advanced prostate cancer. Proc Natl Acad Sci USA. National Academy of Sciences; 2019;116:11428–36.

7. Kumar A, Coleman I, Morrissey C, Zhang X, True LD, Gulati R, et al. Substantial interindividual and limited intraindividual genomic diversity among tumors from men with metastatic prostate cancer. Nat Med. Nature Publishing Group; 2016;22:369–78.

8. Beltran H, Prandi D, Mosquera J-M, Benelli M, Puca L, Cyrta J, et al. Divergent clonal evolution of castration-resistant neuroendocrine prostate cancer. Nat Med. Nature Publishing Group; 2016;22:298–305.
